# Second messenger analogues highlight unexpected substrate sensitivity of CD38: total synthesis of the hybrid “L-cyclic inosine 5′-diphosphate ribose”

**DOI:** 10.1038/s41598-017-16388-0

**Published:** 2017-11-23

**Authors:** Joanna M. Watt, Richard Graeff, Mark P. Thomas, Barry V. L. Potter

**Affiliations:** 1 Wolfson Laboratory of Medicinal Chemistry, Department of Pharmacy and Pharmacology, University of Bath, Claverton Down, Bath, BA2 7AY UK; 2 Department of Physiology, University of Hong Kong, Hong Kong, China; 3 Medicinal Chemistry and Drug Discovery, Department of Pharmacology, University of Oxford, Mansfield Road, Oxford, OX1 3QT UK

## Abstract

The multifunctional, transmembrane glycoprotein human CD38 catalyses the synthesis of three key Ca^2+^-mobilising messengers, including cyclic adenosine 5′-diphosphate ribose (cADPR), and CD38 knockout studies have revealed the relevance of the related signalling pathways to disease. To generate inhibitors of CD38 by total synthesis, analogues based on the cyclic inosine 5′-diphosphate ribose (cIDPR) template were synthesised. In the first example of a sugar hybrid cIDPR analogue, “L-cIDPR”, the natural “northern” *N*1-linked D-ribose of cADPR was replaced by L-ribose. L-cIDPR is surprisingly still hydrolysed by CD38, whereas 8-Br-L-cIDPR is not cleaved, even at high enzyme concentrations. Thus, the inhibitory activity of L-cIDPR analogues appears to depend upon substitution of the base at *C*-8; 8-Br-L-cIDPR and 8-NH_2_-L-cIDPR inhibit CD38-mediated cADPR hydrolysis (IC_50_ 7 μM and 21 µM respectively) with 8-Br-L-cIDPR over 20-fold more potent than 8-Br-cIDPR. In contrast, L-cIDPR displays a comparative 75-fold reduction in activity, but is only *ca* 2-fold less potent than cIDPR itself. Molecular modelling was used to explore the interaction of the CD38 catalytic residue Glu-226 with the “northern” ribose. We propose that Glu226 still acts as the catalytic residue even for an L-sugar substrate. 8-Br-L-cIDPR potentially binds non-productively in an upside-down fashion. Results highlight the key role of the “northern” ribose in the interaction of cADPR with CD38.

## Introduction

**Figure 1:**
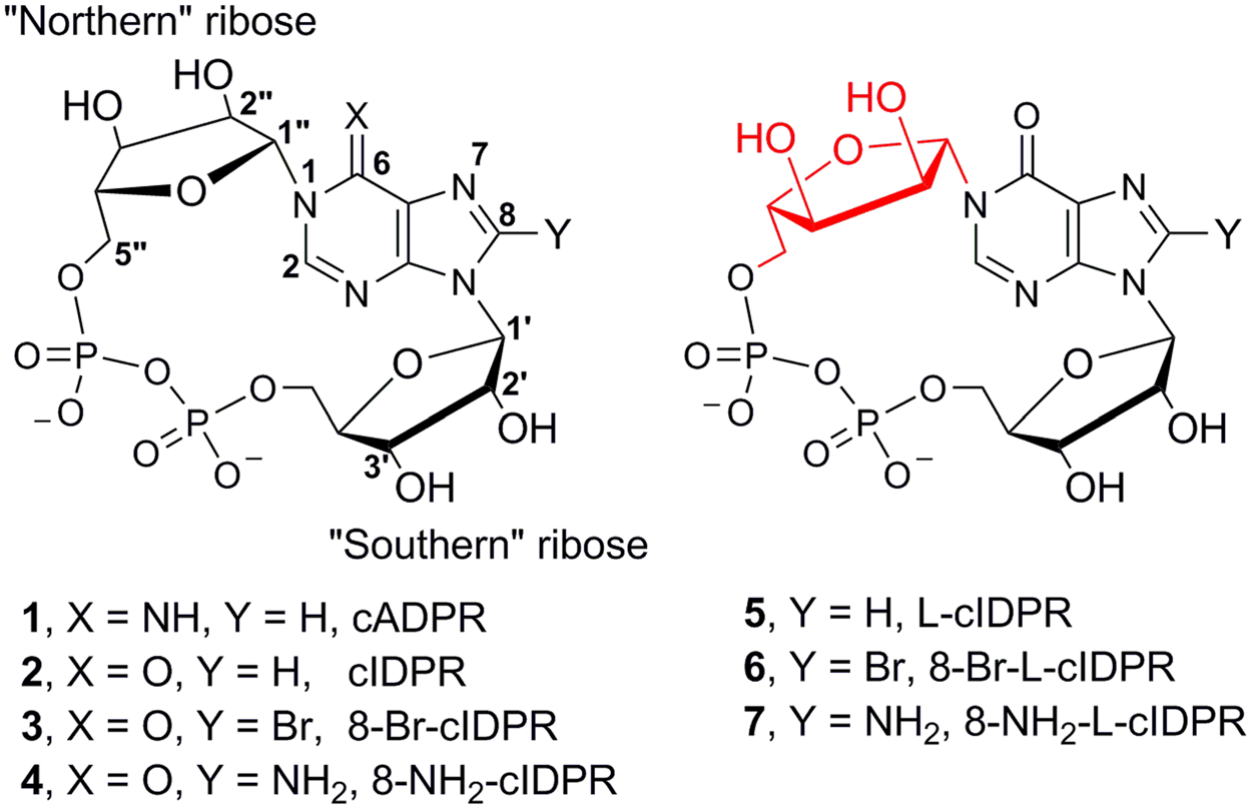
The structure of cADPR, cIDPR and L-cIDPR analogues.

The calcium-releasing second messengers, cyclic adenosine 5′-diphosphate ribose (cADPR, **1**, Fig. 1)^1^ and adenosine 5′-diphosphate ribose (ADPR)^2^ are synthesised in humans by CD38 from nicotinamide adenine dinucleotide (NAD^+^). Under acidic conditions, CD38 can also generate the most potent Ca^2+^-releasing second messenger known to date, nicotinic acid adenine dinucleotide 2′-phosphate (NAADP)^3^, from NADP.

The transmembrane glycoprotein CD38 functions both as a surface receptor in the immune system and a multifunctional ADP-ribosyl cyclase (ADPRC) ectoenzyme. Its catalytic domain may be either extracellular (type II) or intracellular (type III)^4^. We recently confirmed the presence of both CD38 activities in Jurkat T-cells using the non-membrane permeant CD38 inhibitor araF-NAD^5^. CD38 is a marker in AIDS progression^6^ and a negative prognostic marker of chronic lymphocytic leukaemia^7^. The CD38-cADPR pathway is implicated in the pathogenesis of asthma^8^ and Alzheimer’s disease^9^. It acts to regulate intracellular levels of NAD^+^ and therefore is intricately linked to energy homeostasis, signal transduction and aging^10–13^. CD38 is a clinical target for antibody therapy in treating multiple myeloma with encouraging efficacy in patients^14^. Its emerging role in disease states is thus stimulating the search for new CD38 modulators and particularly small molecule inhibitors to provide structural clues for drug design and as potential therapeutic candidates. To date, the reported inhibitors of CD38 are either mechanism-based covalent inhibitors^15^, or reversible, competitive, non-covalent inhibitors. Competitive inhibitors are diverse in structure, including NAD^+^ analogues^16^, flavonoids^17^ and those developed from library hits^18,19^.

cADPR Acts as a principal second messenger, mobilising intracellular calcium^20–23^. We are interested in exploiting the common intermediate in cADPR formation and hydrolysis by CD38^24,25^ using product-like inhibitors. cADPR Analogues have been accessed by either a *chemo-enzymatic* route, modelled on its biosynthesis from NAD^+^, or by total chemical synthesis. *Chemo-enzymatic* routes rely on *Aplysia californica* cyclase recognising an NAD^+^ analogue as a substrate and cyclising at the desired *N*1 position. This limits their use, particularly for analogues that are modified at the locus of the forming *N*1-glycosidic bond [although see^24^]. However, cADPR itself is unattractive for inhibitor design since it is readily hydrolysed at the unstable *N*1 link to give the linear adenosine 5′-diphosphoribose (ADPR), itself a calcium-mobilising second messenger^26–28^. We previously reported a *chemo-enzymatic* route to cyclic inosine 5′-diphosphate ribose via its 8-bromo derivative [*N*1-cIDPR (abbreviated as cIDPR), **2** and 8-Br-cIDPR, **3**, Fig. 1]^29^. Chemically and biologically stable (but *vide infra*), cIDPR and 8-Br-cIDPR both inhibit cADPR hydrolysis by the catalytic domain of CD38 (shCD38; IC_50_ of 276 and 158 µM respectively).

Many total synthetic routes to cADPR analogues required considerable modification of the “northern” ribose in order to generate a stable *N*1-link^30–34^. The difficulty of selective *N*1-ribosylation meant that this tended to be the site at which structural modifications were carried out to overcome issues with synthetic tractability. We developed modified Vorbrüggen conditions that effect completely stereo- and regiospecific introduction of an acetylated ribose at the *N*1-position of a protected inosine^35–37^. Unlike the *chemo-enzymatic* or other synthetic routes, this permits further exploration of the structure-activity relationship at the locus of CD38 catalytic activity using the stable cIDPR template.

Crystallography of shCD38 has identified the mechanism by which NAD^+^ is cyclised to cADPR and ADPR^38^. Glu146 is critical in regulating the multi-functionality of CD38-mediated NADase, ADP-ribosyl cyclase and cADPR hydrolysis activities and Glu226 is the catalytic residue, since its mutation essentially eliminates catalytic activity^39^. Crystal structures obtained with shCD38 and cADPR analogues^40,41^ suggest that the “northern” ribose monophosphate region is highly conserved. In the catalytic site, cADPR forms two hydrogen bonds through *N*6 and *N*7 to Glu146 and interacts with Glu226 through the 2″- and 3″-OH of the “northern” ribose. Taken together, this suggests a critical role for the base and “northern” ribose in the binding of cADPR analogues to CD38, as might be predicted for the locus of both cADPR formation and degradation. We have previously shown that small fragments consisting of only these elements (*N*1-inosine 5′-monophosphates, *N*1-IMP), or analogues of cIDPR with the “southern” ribose replaced by a butyl linker, inhibit cADPR hydrolysis by shCD38 with IC_50_ values in the low µM region^36,41^. Crystallography of a hydrolysed cADPR analogue revealed that interactions with Glu146 and Glu226 were maintained even after hydrolysis of the *N*1-glycosidic bond^36^.

cADPR possesses two ribose sugar motifs and both of these are of the D-configuration. Analogues where the ribose is substituted for an alternative sugar have not previously been explored. Therefore, we designed and synthesised the hybrid cIDPR analogue “L-cIDPR”, where the unnatural sugar enantiomer L-ribose is employed to generate an analogue that presents the “northern” ribose hydroxyl groups in a different spatial arrangement. We hoped that an L-configuration in an analogue at this site would probe the interaction between the hydroxyl groups of the “northern” ribose and the catalytic residue Glu226 of shCD38, and predicted that modification of this critical interaction might affect binding affinity.

We report here the synthesis of L-cIDPR and its evaluation as an inhibitor of cADPR hydrolysis by shCD38. L-cIDPR is surprisingly able to access the catalytic machinery of shCD38, being hydrolysed at high enzyme concentrations. We also report the synthesis of further base-modified analogues of L-cIDPR and their activity as inhibitors of cADPR hydrolysis by shCD38 and HPLC studies to examine the stability of the novel analogues towards hydrolysis by shCD38.

## Results and Discussion

### Synthesis of L-ribose cIDPR analogues

**Figure 2:**
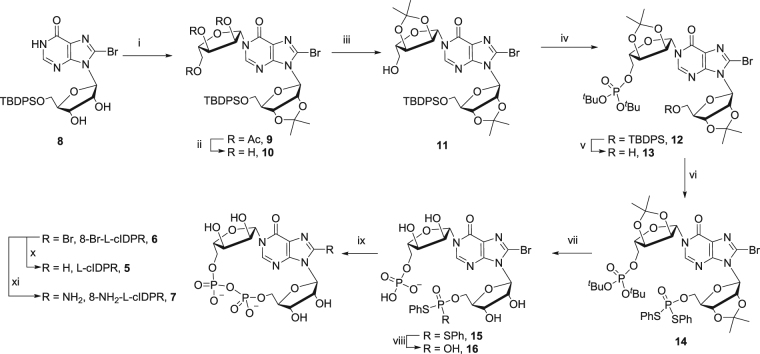
Synthesis of L-ribose cIDPR analogues. Reagents i) (**a**) DBU, DCM (**b**) Tetraacetyl-L-ribose, TMSOTf, 85%; ii) NH_3_, MeOH, 100%; iii) pTsOH, H_3_CC(OMe)_2_CH_3_, Acetone, 100%; iv) (**a**) (^t^BuO)_2_PN(^i^Pr)_2_, 5-Ph-1H-tetrazole, DCM (**b**) H_2_O_2_, Et_3_N, 60%; v) TBAF.3H_2_O, AcOH, 96%; vi) PSS, TPS-Cl, 5-Ph-1H-tetrazole, pyridine, 78%; vii) 50% TFA (aq.), 70%; viii) 0.1 M NaOH-dioxane; ix) I_2_, 3 Å MS, pyridine, 18% over 2 steps; x) H_2_, Pd/C, NaHCO_3_, EtOH-H_2_O, 46%; xi) (**a**) TMSN_3_, DMF, (**b**) Dithiothreitol, 0.05 M TEAB, 23%.

Starting from 5′-*O*-(*tert*-butyldiphenylsilyl)-2′,3′-*O*-isopropylidene-8-bromoinosine^35^ (**8**, Fig. 2) we introduced 2,3,5-*O*-triacetyl-β-L-ribofuranose by *N*1-ribosylation. The optimised reaction conditions for stereo- and regio-selective *N*1-coupling gave the desired product **9** in 85% yield, with no trace of the unwanted *O*-6 regioisomer. The 5″-OH was then revealed by sequential deprotection of the triol using methanolic ammonia and reprotection of the 2″,3″-diol was accomplished as its isopropylidene ketal. Treatment of **11** with *N,N*-diisopropyldibenzylphosphoramidite and 5-phenyl-1-*H*-tetrazole generated the 5″-*O*-phosphite, which was oxidised under basic conditions using H_2_O_2_ and triethylamine to generate **12**. Deprotection of the 5′-*O*-TBDPS ether was carried out under neutral conditions using tetrabutylammonium fluoride (TBAF) and acetic acid to prepare **13**. The second protected phosphate was introduced at the 5′-OH using *S,S*-diphenylphosphorodithioate (PSS), 2,4,6-triisopropylbenzenesulfonyl chloride (TPS-Cl) and 5-phenyl-1*H*-tetrazole to generate **14**. Sequential deprotection of the phosphates was carried out using 50% trifluoroacetic acid (TFA) to remove the *tert*-butyl phosphate esters from the “northern” ribose and the two isopropylidene groups, then 0.1 M NaOH in dioxane to selectively deprotect one thiophenyl ester from the “southern” ribose. Cyclisation of **16** was carried out using iodine and 3 Å molecular sieves in pyridine under dilute conditions to prepare 8-Br-L-cIDPR **6** (Note: The notation ‘L’ refers to the stereochemistry of the “northern” ribose only and is not systematic.). The 8-bromo substituent was removed using Pd/C catalysis under an atmosphere of H_2_ to prepare the L-cIDPR analogue **5**. Treatment of **6** with trimethylsilyl azide (TMSN_3_) in DMF was monitored by RP-HPLC (λ = 255 nm → 277 nm) and the crude material treated with dithiothreitol to generate 8-amino L-ribose cIDPR, **7**.

### Inhibition of cADPR hydrolysis by CD38

**Figure 3:**
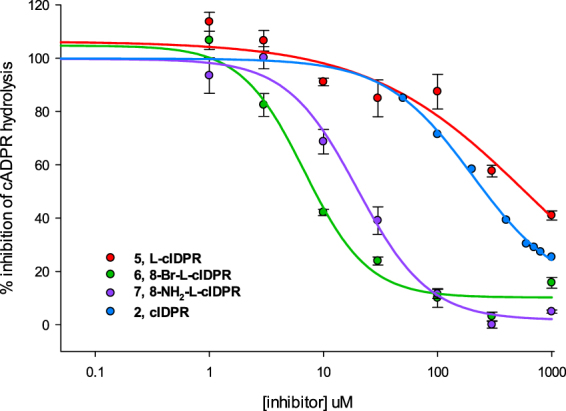
Inhibition of shCD38-mediated cADPR hydrolysis by analogues **5-7**.

The ability of compounds **5**-**7** to inhibit hydrolysis of cADPR by human shCD38 was evaluated by a fluorimetric cycling assay^42^. The analogues inhibit hydrolysis of cADPR in a concentration-dependent manner (Fig. 3). 8-Br-L-cIDPR **6** and 8-NH_2_-L-cIDPR **7** are good inhibitors, with IC_50_ values of 7 μM and 21 μM respectively. In contrast, the parent compound, L-cIDPR **5**, is a poor inhibitor with IC_50_ of 526 μM, Table 1.

**Table 1:** Half maximal values for inhibition of cADPR hydrolysis by shCD38.

	D-Ribose		L-Ribose	
	cIDPR	IC_50_ (μM)^41^	L-cIDPR	IC_50_ (μM)
8-H	**2**	276 ± 10	**5**	526 ± 36
8-Br	**3**	158 ± 13	**6**	7 ± 1
8-NH_2_	**4**	56 ± 9	**7**	21 ± 2

The contrasting inhibitory activity upon substitution of the “northern” D-ribose by L-ribose was unexpected. L-cIDPR **5** is a roughly twofold poorer inhibitor of hydrolysis than cIDPR – which could be ascribed to changes within the “northern” ribose hydroxyl stereochemistry, meaning that critical interactions with the binding pocket are not fully realised. This might be anticipated *prima facie*, but is nevertheless somewhat surprising given the apparent stereochemical insult afforded by the L-sugar to the CD38 enzymatic machinery. Astonishingly, by comparison, 8-Br-L-cIDPR **6** is highly active with an IC_50_ of 7 μM. We had already seen that substitution of cIDPR to generate 8-Br-cIDPR improves the inhibitory activity of this analogue from 276 μM to 158 μM^41^; however, addition of the 8-bromo atom when the “northern” ribose is in the L-configuration generates a 75-fold increase in inhibitory activity (from 526 μM to 7 μM). In our previous studies, the introduction of an 8-NH_2_ substituent on the adenine ring resulted in a further improvement of inhibitory activity (approximately 5-fold increase compared to the parent molecule and 3-fold increase compared to the 8-Br substituted analogue). This increase in binding affinity was shown, by modelling and crystallography, to result from an additional interaction with Asp-155^41^. A comparable trend was not observed for the L-ribose cIDPR analogues prepared here, and this may suggest that the binding of these analogues has changed to adapt to the L-ribose substitution.

### Conformational analysis

**Figure 4:**
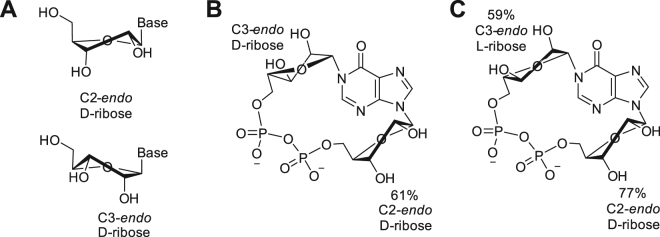
(**A**) Schematic representation of the ribofuranose ring in both C2-*endo* and C3-*endo* conformations; (**B**) From ^1^H-NMR data, cIDPR (**2**) in solution is predicted to display a C3-*endo* configuration in the “northern” ribose and 61% C2-*endo* configuration in the “southern” ribose; (**C**) L-cIDPR (**5**) is predicted to display a 59% C3-*endo* and 77% C2-*endo* configuration, respectively.

In solution, the ribose ring of nucleosides and nucleotides exists in conformational equilibrium between the C2′-*endo* and C3′-*endo* forms. As illustrated in Fig. 4A, the particular conformation adopted affects the spatial presentation of the hydroxyl groups and consequently would be expected to affect the interaction of a ligand with the binding pocket. Indeed, the conformation adopted by the southern ribose in cADPR analogues was shown to underpin their activity at the sea urchin cADPR receptor^43^. Using the method established by Altona and Sundaralingham^44^, the ratio of C2′-*endo*/C3′-*endo* forms may be mathematically calculated from the observed coupling constants in the ^1^H-NMR spectrum.

We used the ^1^H-NMR spectra of analogues **5-7** to determine the *syn/anti*-conformation of the purine base^45^ relative to the *N*9-ribose and to examine the ribose ring pucker of both the *N*9 and *N*1 sugars, Table 2.

**Table 2:** Predicted conformations of L-ribose analogues **5-7**.

	Nucleoside				*N*9-ribose			*N*1-ribose		
	H-1′	H-2′	Δ_H-1′-H-2′_	Conf.	*J*_1′-2′_	*J*_3′-4′_	C_2_-endo	*J*_1″-2″_	*J*_3″-4″_	C_2_-endo
L-cIDPR, **5**	5.93	5.09	0.84	*Syn*	6.5	1.9	77%	3.5	5.0	41%
8-Br-L-cIDPR, **6**	6.05	5.15	0.9	*Syn*	6.8	1.8	79%	3.8	*n.d*.	38%
8-NH_2_-L-cIDPR, **7**	5.90	5.30	0.6	*Syn*	6.7	*n.d*.	67%	3.5	*n.d*.	35%
cIDPR, **2** ^29^	5.89	5.18	0.71	*Syn*	6.1	*n.d*.	61%	—		0%

As would be expected for conformationally restricted, cyclised analogues with two intact riboses, all three analogues adopt a *syn* conformation. Analysis of the *N*9-ribose conformation predicted that **5**-**7** would adopt a primarily C2-*endo* ring pucker of the “southern” ribose in free solution – matching that of cIDPR. For the “northern” *N*1-ribose, the three L-ribose analogues are predicted to have a predominantly C3-*endo* conformation, calculated using the coupling constant between H-1″ and H-2″ whereas cIDPR displays only a singlet for H-1″, suggesting a dihedral angle of 90° and a C3-*endo* conformation. The effect of the predominant conformation on 2″- and 3″-hydroxyl group orientation is illustrated for cIDPR (Fig. 4B) and L-cIDPR (Fig. 4C).

**Figure 5:**
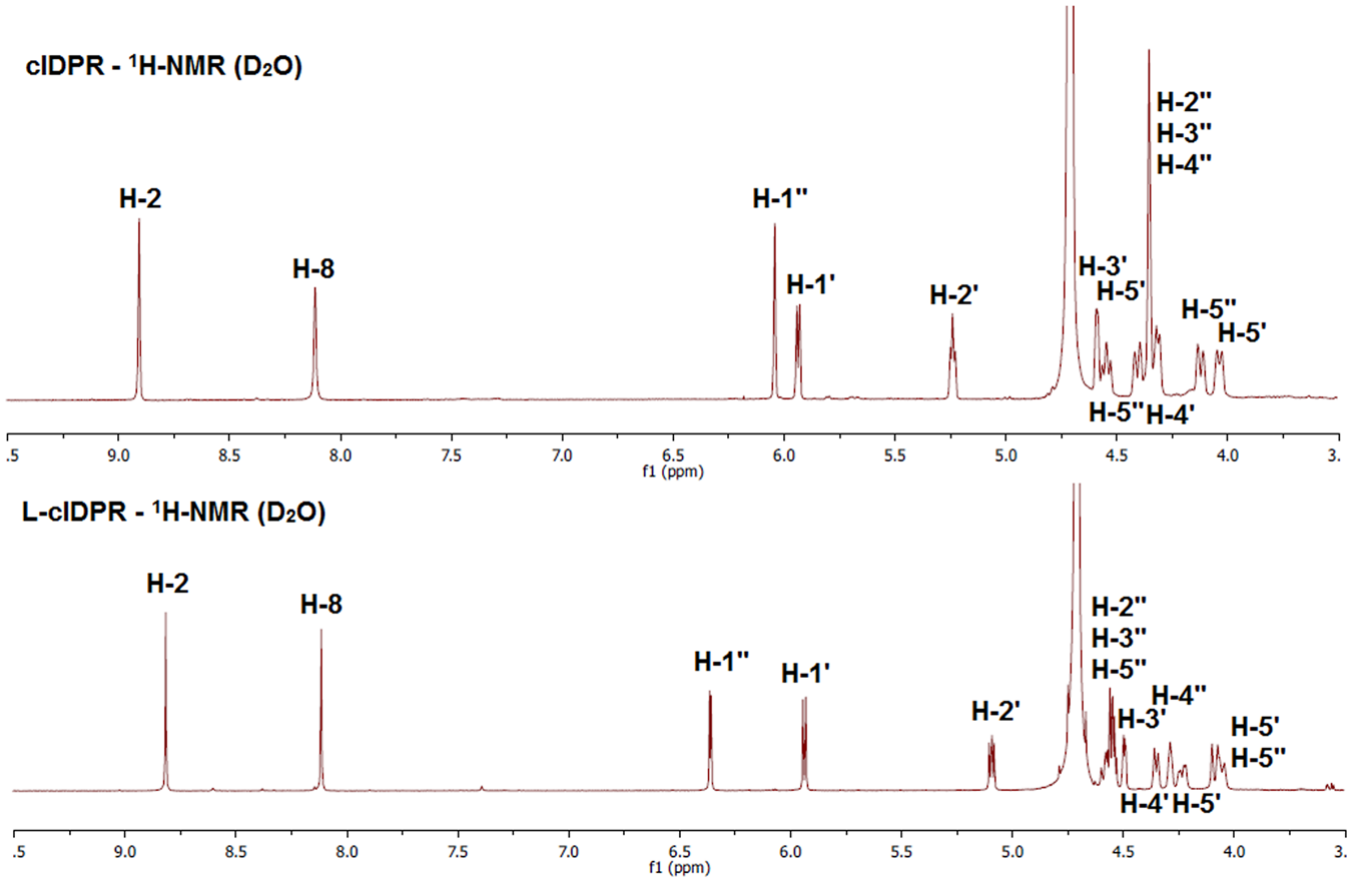
Comparison of the sugar region (6.5-3.5 ppm) of the 500 MHz ^1^H-NMR spectrum of cIDPR and L-cIDPR.

The “northern” ribose anomeric proton of L-cIDPR is shifted downfield by 0.3 ppm compared to cIDPR, suggesting it is more deshielded and the ring protons H-2″-4″ shifted upfield by 0.2 ppm. These changes reflect the different environments occupied by the ring protons and hydroxyl groups as a result of the change in sugar stereochemistry (Fig. 5).

## HPLC studies

We recently discovered^36^ that high concentrations of shCD38 were in fact able to hydrolyse the cIDPR scaffold, when crystallography of 8-NH_2_-*N*9-butyl-cIDPR revealed an *N*1-hydrolysed product in the active site. This was somewhat surprising, as initial studies at lower concentrations had led to the cIDPR template being described as non-hydrolysable^46^. Hydrolysis would however not be predicted to occur at the concentrations found in enzyme assays, or those likely under physiological conditions (Supplementary Fig. S1, top two panels), so the cIDPR template can still be assumed to be biologically stable. Study of cIDPR analogues under these conditions can thus be used to evaluate the ability of the enzyme to turn over an analogue, and of an analogue to access the CD38 catalytic machinery. Interestingly, cIDPR is also enzymatically hydrolysable by the related *Aplysia* cyclase under extreme conditions, as revealed by X-ray crystallographic studies. Incubation of 30 mM cIDPR with wild-type *Aplysia* cyclase showed that cIDPR was clearly hydrolysed to IDPR at the *N*1 bond. In this instance, both linear IDPR and the cyclic *N*1-cIDPR are present and close to each other, with cIDPR in an upside-down orientation compared to cIDPR in CD38 (Q Liu, C Moreau, Q Hao, B V L Potter unpublished data, Supplementary Figure S2). Such an upside down binding mode for cIDPR in the *Aplysia* enzyme had also been observed earlier^47^. However, cIDPR bound in this way is clearly not an active conformation for cleavage in relation to the known catalytic residue and therefore this mode would be likely to lead to enzyme inhibition.

**Figure 6:**
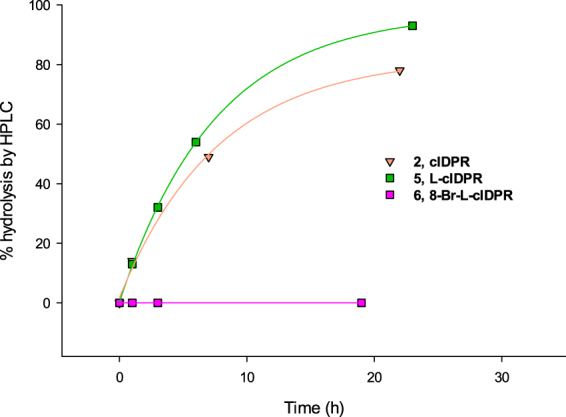
HPLC studies of cIDPR and novel analogues with shCD38.

Incubation of **5** (1 mM final concentration) with 4 mg/mL shCD38 [enzyme in heavy excess] was monitored using RP-HPLC. The peak corresponding to **5** (R_T_ = 12.5 min) reduced in intensity over time, alongside the appearance of a new peak (R_T =_ 15.6 min), that was characteristic of an ADPR analogue (Fig. 6, Supplementary Fig. S1 third panel). No change in the original peak was observed in a parallel control experiment containing no shCD38. The rate of hydrolysis was similar, or slightly faster than that observed for cIDPR (**2**) under the same conditions. Surprisingly, treatment of analogue **6** under the same conditions, resulted in no hydrolysis, even after 22 hours (Fig. 6 and Supplementary Fig. S1, lower panel).

The observed hydrolysis of L-cIDPR at high concentrations of shCD38 demonstrates the flexibility of the CD38 catalytic machinery. The inability of CD38 to hydrolyse 8-Br-L-cIDPR perhaps suggests that this ligand adopts a different binding pose, presumably due to its additional 8-Br substituent combined with the change in hydroxyl stereochemistry resulting from introduction of the L-ribose at *N*1. Such a change in binding mode would help rationalise the observed difference in inhibition of CD38-mediated cADPR hydrolysis. It would seem feasible that 8-Br-L-cIDPR could be an excellent inhibitor of CD38 and not a substrate by virtue of binding in an upside-down fashion. Such a mode for a hybrid “northern” L-ribose analogue would put the “southern” D-ribose in a pseudo-northern ribose position, and the L-ribose in a pseudo-southern ribose position. We know from previous studies that the southern ribose is not essential for CD38 activity and can be replaced^36^. Unfortunately, attempts to crystallise 8-Br-L-cIDPR with CD38 to determine the exact binding pose were not successful.

## Molecular Modelling

Docking of the “northern” ribose modified analogues using the modelling software GOLD generated several different poses in the binding site. No pose, by either visual inspection or by comparison of the docking scores, stood out as being obviously better than any of the others. As an alternative approach, the modified ligands were prepared by amending the stereochemistry of the natural “northern” ribose of cIDPR in the published 2PGJ and 3U4H crystal structures. The 8-substituent was also altered accordingly to 8-H, 8-Br, or 8-NH_2_, to generate a set of ligands in both crystal structures. The protein-ligand complexes were minimised and the binding interactions compared. It is important to note that the latter method is limited, in that it assumes that the L-ribose compounds bind to CD38 with the same basic pose as the natural D-ribose compounds and we would not be able to identify any potential completely different binding poses adopted by the L-cIDPR ligands. However, the approach is justified by the fact that L-ribose cIDPR must be able to access the catalytic residue of CD38 to be a substrate.

**Figure 7:**
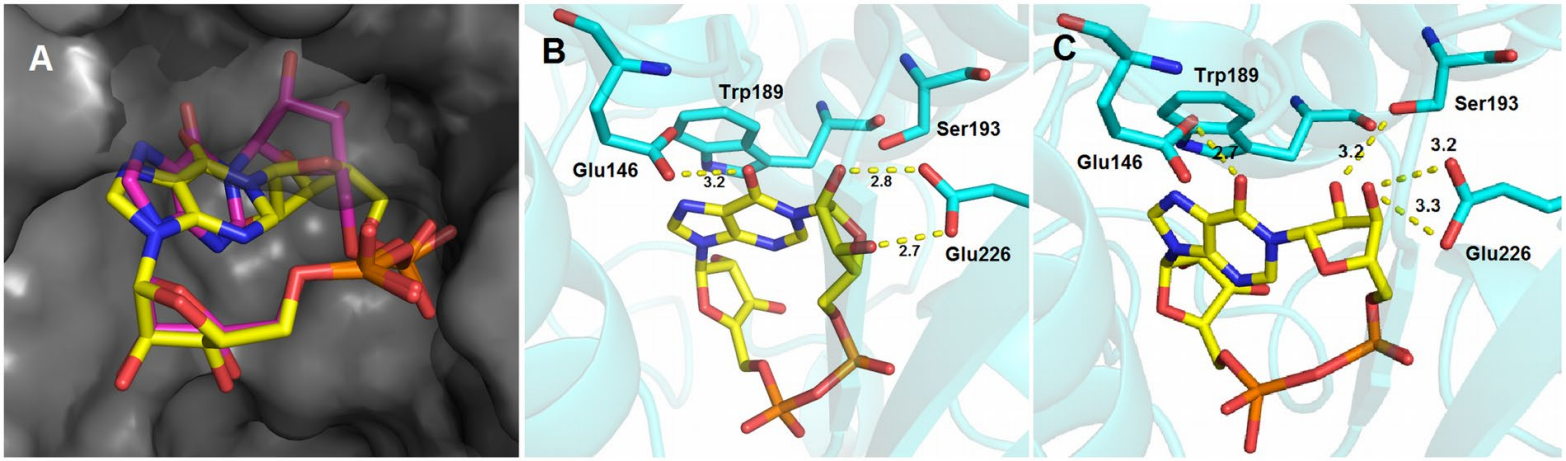
(**A**) cIDPR (pink) and minimised L-cIDPR (yellow) in the 2PGJ crystal structure. Hypoxanthine ring tilt and good overlay of the southern ribose and pyrophosphate regions; (**B**) cIDPR (yellow) crystal structure indicates H-bonds from 2″- and 3″-OH to Glu226, stacking of hypoxanthine ring with Trp189, H-bond from 6 = O to Glu146; (**C**) L-cIDPR (yellow) minimised structure suggests disruption of π-stacking with Trp189 and significant rotation of the L-ribose to maintain at least one H-bond with Glu226 and potentially form a second H-bond between the 2″-OH and Ser-193. H-bonds are shown as yellow dashed lines with distances labelled in Å, interacting residues as shown as cyan sticks.

All analogues had similar poses to cIDPR after minimisation, and showed good overlap of the southern ribose sugar and pyrophosphate backbone. The L-configuration “northern” ribose is rotated around its *N*1-*C*1 and *C*4-*C*5 bonds, placing the furanose ring oxygen in the centre of the macrocycle and directing the two ring hydroxyl groups towards the binding pocket. The 2″- and 3″-hydroxyl group orientation is significantly changed due to the altered “northern” ribose stereochemistry, and without this rotation, they would point towards the centre of the binding pocket and not be able to contribute to binding. Therefore, the rotation of the ribose maximises the possibility of H-bonds to the catalytic residue Glu226 (Fig. 7).

**Figure 8:**
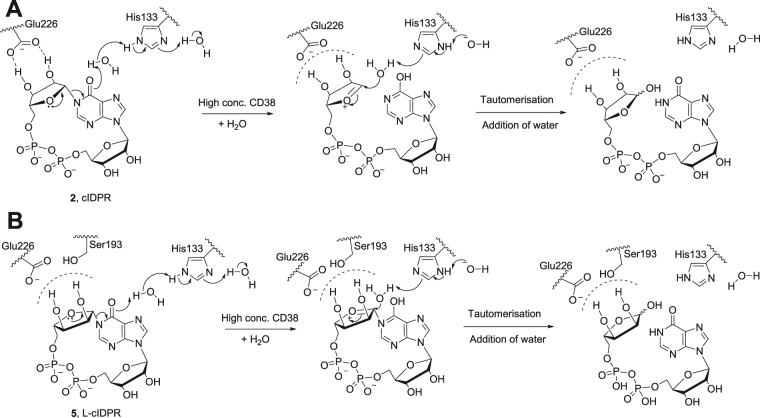
Schematic representing (**A**) cIDPR in the active site (from 2JPG) and active site residues proposed to lead to hydrolysis^36^; (**B**) L-cIDPR in the active site – twisted base and non-natural ribose sugar may alter catalysis in the active site. Possible additional interaction with Ser193 in the binding site is predicted by modelling.

In all the L-ribose ligand complexes we generated, the L-ribose linked hypoxanthine ring is tilted, suggesting that these ligands may have disrupted π-stacking with Trp189 (Fig. 7C). The potential interaction of the L-ribose itself with the catalytic residue Glu226 is very interesting; the altered stereochemistry of the L-configuration ribose appears to prevent binding of both 2″-OH and 3″-OH to this residue meaning that only one H-bond is formed with Glu226 and the 3″-OH. However, the 2″-OH in the L-ribose conformation is correctly aligned to make a new H-bond with Ser193 (Fig. 7C). This suggests that the catalytically active conformation of L-cIDPR that allows scission of the *N*1-bond in the active site may differ from cIDPR (Fig. 8). Nevertheless, it is anticipated that only relatively minimal movement of the critical residue Glu226 should be required to accommodate an interaction with the L-ribose hydroxyls and facilitation of attack at the glycosidic bond, in comparison to the natural D-isomer. It is worth noting the immense value of L-nucleosides and derivatives such as Lamivudine, approved against HIV and HBV, that have been shown to have profound antiviral activity with many analogues in clinical trials and abundant evidence for their inhibition of and processing by host and viral enzymes^48^.

We have previously predicted and confirmed by crystallography that the introduction of an 8-NH_2_-substituent can introduce an additional H-bond to Asp-155 in the shCD38 binding pocket^41^. For the 8-substituted L-cIDPR analogues our modelling suggested that the tilt of the hypoxanthine ring to accommodate the rotation of the L-configuration *N*1*-*ribose does not interfere with the H-bond to Asp-155 (Supplementary Figure S3). These observations give some rationale to the improved affinity of both 8-Br-L-cIDPR and 8-NH_2_-cIDPR compared to L-cIDPR but seem unlikely to be solely responsible for the extent of the difference in inhibitory activity that is observed.

## Conclusion

Synthesis of the proposed ligands was facilitated by selective *N*1-glycosylation, and three hybrid L-ribose cIDPR-based analogues, substituted in the 8-position of adenine were prepared. Such analogues are unlikely to be accessible via a *chemo-enzymatic* route and illustrate the utility of our recently developed *N*1-ribosylation strategy to explore modifications to this sensitive region where both cyclisation and hydrolysis takes place. 8-Br-L-cIDPR is a potent (7 μM) inhibitor of cADPR hydrolysis by CD38. 8-NH_2_-L-cIDPR is similar in potency (21 μM), not showing the enhanced activity relative to its parent that has been previously observed with an extra 8-amino group for another analogue series^41^. Most surprising was the comparable lack of inhibitory activity of the parent compound, L-cIDPR, and its hydrolysis by CD38, albeit at high enzyme concentrations, showing that this analogue can still access the catalytic machinery. In contrast, 8-bromo-L-cIDPR was unaffected by high concentrations of CD38. In our past studies with cADPR analogues docking to CD38 we have been able to employ GOLD to reproduce very accurately the observed crystallographic binding modes, both with a compound from the inosine-based template, 8-amino-cIDPR, and also with another based upon cADPR itself, the carbocylic analogue cADPcR^41^. Docking studies with the L-ribose-modified ligands based on published crystal structures, however, struggled to predict clear preferred poses for such compounds with the catalytic site, highlighting potentially the critical nature of the stereochemical insult afforded by the L-ribose sugar. Adapting the natural ligand in the active site suggested that interactions could be maintained with the catalytic residue Glu226 by twisting the hypoxanthine ring in the binding pocket and this is supported by the substrate activity of L-cIDPR. It seems feasible, however, that the resistance to hydrolysis of the 8-bromo-L-cIDPR could be ascribed to a non-productive binding mode, possibly one in an upside-down configuration. Taken together, the analogues prepared in this study demonstrate that the “northern” ribose is an area that is very sensitive to modification, presents key binding partners for CD38 and offers some surprising insights.

## Experimental Section

### General

All reagents and solvents were of commercial quality and were used without further purification, unless described otherwise. The human CD38 catalytic domain (shCD38) was expressed and purified as described previously^36,39^. Unless otherwise stated, all reactions were carried out under an inert atmosphere of argon. ^1^H, ^13^C, and ^31^P NMR spectra were collected on a Jeol Delta 270 MHz, Varian Mercury 400 MHz or Bruker Avance III 500 MHz Spectrometer. All ^1^H- and ^13^C-NMR assignments are based on *g*COSY, *g*HMBC, *g*HSQC, and DEPT-135 experiments. Abbreviations for splitting patterns are as follows: b, broad; s, singlet; d, doublet; t, triplet; m, multiplet etc. Coupling constants are given in Hertz (Hz). High resolution time-of-flight mass spectra were obtained on a Bruker Daltonics micrOTOF mass spectrometer using electrospray ionisation (ESI). Analytical HPLC analyses were carried out on a Waters 2695 Alliance module equipped with a Waters 2996 Photodiode Array Detector (210–350 nm). The chromatographic system consisted of a Hichrom Guard Column for HPLC and a Phenomenex Synergi 4μ MAX-RP 80 A column (150 × 4.60 mm), eluted at 1 mL/min with the following ion-pair buffer: 0.17% (m/v) cetrimide and 45% (v/v) phosphate buffer (pH 6.4) in MeOH. Synthetic phosphates were assayed and quantified by Ames phosphate test^49^.

### *N*1-(2′′,3′′,5′′-Tri-*O*-acetyl-β-L-ribofuranosyl)-5′-*O*-(*tert*-butyldiphenylsilyl)-2′,3′-*O*-isopropylidene-8-bromoinosine (9)

5′-*O*-TBDPS-2′,3′-*O*-isopropylidene-8-bromoinosine **8** ^35^ (980 mg, 1.57 mmol) was taken up in DCM (10.0 mL) and DBU (703 µL, 4.70 mmol) added. After 30 minutes, 1,2,3,5-tetra-*O*-acetyl-β-L-ribofuranose (548 mg, 1.72 mmol) was added and the solution cooled to -78 °C. Trimethylsilyl trifluoromethanesulfonate (1.13 mL, 6.26 mmol) was added dropwise and the solution stirred for a further 45 min before warming to rt. After 2.5 H, NaHCO_3_ (sat. aq.) was added and the crude material extracted into DCM (×3). The combined organic fractions were dried (Na_2_SO_4_), and solvent evaporated under reduced pressure. The residue was purified by column chromatography on silica gel eluting with PE:EtOAc (1:0 → 0:1 v/v) to afford the *title compound* (1.18 g, 85%) as a colourless glass; *R*_f_ = 0.69 (EtOAc:PE 3:1 v/v); ^1^H-NMR (400 MHz, CDCl_3_) δ 7.96 (s, 1H, H-2), 7.57 (dd, 2H, *J* = 8.0, 1.4), 7.53 (dd, 2H, *J* = 8.0, 1.4), 7.37-7.28 (m, 4H), 7.23 (t, 2H, *J* = 8.0), 6.33 (d, 1H, *J* = 4.2, H-1′′), 6.14 (d, 1H, *J* = 2.2, H-1′), 5.45 (dd, 1H, *J* = 6.4, 2.2, H-2′), 5.42-5.37 (m, 2H, H-2′′ and H-3′′), 5.00 (dd, 1H, *J* = 6.4, 4.2, H-3′), 4.41 (dd, 1H, *J* = 4.9, 3.8, H-4′′), 4.37-4.36 (m, 2H, 2 ×H-5′′), 4.31 (dd, 1H, *J* = 4.2, 1.4, H-4′), 3.83 (dd, 1H, *J* = 11.0, 5.2, H-5′a), 3.74 (dd, 1H, *J* = 11.0, 6.6, H-5′b), 2.11, 2.08, 2.07 (each s, 3H, 3 × OAc), 1.61 (s, 3H, CH_3_), 1.37 (s, 3H, CH_3_), 1.00 (s, 9H, ^*t*^Bu) ppm; ^13^C-NMR (100 MHz, CDCl_3_) 169.9, 169.5, 169.4, 154.5, 147.6, 143.8, 135.43 (2C), 135.42 (2C), 133.3, 132.8, 129.6 (2C), 127.5 (2C), 127.4 (2C), 126.3, 124.7, 114.5, 90.9, 87.7, 87.1, 83.3, 81.3, 80.0, 74.1, 69.9, 63.9, 62.8, 27.2, 26.7 (3C), 25.4, 20.6, 20.4, 20.3, 19.1 ppm; HRMS (ESI^+^) found *m/z* [M + Na]^+^ 905.2013, 907.1994; C_40_H_47_N_4_O_12_^79^BrSiNa requires 905.2035, C_40_H_47_N_4_O_12_^81^BrSiNa requires 907.2015.

### *N*1-(β-L-ribofuranosyl)-5′-*O*-(*tert*-butyldiphenylsilyl)-2′,3′-*O*-isopropylidene-8-bromoinosine (10)

Intermediate **9** (3.56 g, 4.03 mmol) was taken up in MeOH (20 mL) in a pressure tube. The solution was saturated with NH_3_ (g) at 0 °C, then stirred at rt for 12H. The solvent was evaporated and the residue purified by column chromatography on silica gel eluting with DCM:Acetone (1:0 → 0:1 v/v) to afford the *title compound* (3.05 g, 100%) as an off-white amorphous solid; *R*_f_ = 0.24 (PE:EtOAc 1:3 v/v); ^1^H-NMR (400 MHz, CDCl_3_) δ 8.13 (s, 1H, H-2), 7.58 (dd, 2H, *J* = 8.0, 1.3), 7.52 (dd, 2H, *J* = 8.0, 1.3), 7.40-7.31 (m, 4H), 7.22 (t, 2H, *J* = 7.1), 6.12 (d, 1H, *J* = 2.2, H-1′), 5.87 (d, 1H, *J* = 4.1, H-1′′), 5.49 (dd, 1H, *J* = 6.3, 2.2, H-2′), 5.03 (dd, 1H, *J* = 6.3, 3.8, H-3′), 4.78 (d, 1H, *J* = 2.9, 2′′-OH), 4.49-4.46 (m, 1H, H-2′′), 4.42-4.39 (m, 1H, H-3′′), 4.36-4.32 (m, 1H, H-4′), 4.31-4.28 (m, 1H, H-4′′), 3.99-3.93 (m, 1H, H-5′′a), 3.93-3.75 (m, 2H, H-5′a and H-5′′b), 3.73-3.70 (m, 1H, H-5′b), 3.65 (d, 1H, *J* = 4.0, 3′′-OH), 2.62 (bs, 1H, 5′′-OH), 1.60 (s, 3H, CH_3_), 1.37 (s, 3H, CH_3_), 1.00 (s, 9H, ^*t*^Bu) ppm; ^13^C-NMR (100 MHz, CDCl_3_) δ 156.0, 148.4, 144.6, 135.5 (2C), 135.4 (2C), 133.3, 132.9, 129.7, 129.6, 127.6 (2C), 127.4 (2C), 126.8, 124.6, 114.4, 93.9, 91.3, 87.8, 86.4, 83.1, 81.6, 75.6, 70.8, 63.9, 61.9, 27.2, 26.7 (3C), 25.5, 19.1 ppm; HRMS (ESI^+^) found *m/z* [M + Na]^+^ 779.1701 and 781.1700, C_34_H_41_N_4_NaO_9_^79^BrSi requires 779.1718, C_34_H_41_N_4_NaO_9_^81^BrSi requires 781.1698.

### *N*1-(2′′,3′′-*O*-isopropylidene-β-L-ribofuranosyl)-5′-*O*-(*tert*-butyldiphenylsilyl)-2′,3′-*O*-isopropylidene-8-bromoinosine (11)

*p*-TsOH (116 mg, 0.61 mmol) was added to **10** (460 mg, 0.61 mmol) in acetone-2,2-dimethoxypropane (10 mL, 4:1 v/v). After 45 min, DCM and NaHCO_3_ (sat. aq.) were added, and the organic layer dried (Na_2_SO_4_) and evaporated. The residue was taken up in MeOH (5 mL) and DOWEX 50WX8H^+^ resin (15 mg) added to convert any unwanted 5″-*O*-hemi-acetal side product into product. After 30 min the resin was removed by filtration under gravity and the solvent evaporated to obtain the *title compound* (484 mg, 100%) as a white foam; *R*_f_ = 0.74 (DCM:Acetone 1:1 v/v); ^1^H-NMR (400 MHz, CDCl_3_) δ 7.62 (dd, 2H, *J* = 7.0, 1.4), 7.53 (s, 1H, H-2), 7.52 (dd, 2H, *J* = 7.0, 1.4), 7.42-7.32 (m, 4H), 7.22 (t, 2H, *J* = 5.8), 6.12 (d, 1H, *J* = 2.2, H-1′), 5.55 (d, 1H, *J* = 2.6, H-1′′), 5.44 (dd, 1H, *J* = 6.4, 2.2, H-2′), 5.18 (dd, 1H, *J* = 6.5, 2.6, H-2′′), 5.11 (dd, 1H, *J* = 6.5, 3.6, H-3′′), 5.04 (dd, 1H, *J* = 6.4, 3.8, H-3′), 4.36-4.32 (m, 2H, H-4′ and H-4′′), 3.89-3.80 (m, 2 H, H-5′′a and H-5′′b), 3.83 (dd, 1H, *J* = 10.8, 5.7, H-5′a), 3.77 (dd, 1H, *J* = 10.8, 6.2, H-5′b), 3.08 (bd, 1H, *J* = 4.2, 5′′-OH), 1.61 (s, 6 H, 2 × CH_3_), 1.37 (s, 6H, 2 × CH_3_), 1.01 (s, 9H, ^*t*^Bu) ppm; ^13^C-NMR (100 MHz, CDCl_3_) δ 155.0, 148.0, 146.2, 135.5 (2 C), 135.4 (2C), 133.5, 132.9, 129.7 (2C), 127.6 (2C), 127.5 (2C), 126.7, 125.5, 114.4, 114.2, 96.9, 91.3, 88.2, 87.7, 83.6, 83.2, 81.5, 80.6, 63.8, 62.8, 27.3, 27.2, 26.7 (3C), 25.5, 25.2, 19.2 ppm; HRMS (ESI^+^) found *m/z* [M + Na]^+^ 819.2005 and 821.1993; C_37_H_45_N_4_O_9_^79^BrSiNa requires 819.2031, C_37_H_45_N_4_O_9_^81^BrSiNa requires 821.2011.

### *N*1-[2′′,3′′-*O*-isopropylidene-5′′-*O*-(di-*tert-*butyl)-phosphoryl-β-L-ribofuranosyl]-5′-*O*-(*tert*-butyldiphenylsilyl)-2′,3′-*O*-isopropylidene-8-bromoinosine (12)

5-Phenyl-1*H*-tetrazole (425 mg, 2.46 mmol) and *N,N-*diisopropyldibutylphosphoramidite (584 µL, 1.85 mmol) were added to a solution of **11** (985 mg, 1.23 mmol) in DCM (10 mL). After 2H, the solution was cooled to 0 °C and Et_3_N (1.03 µL, 7.38 mmol) and H_2_O_2_ (35%, 269 µL, 3.08 mmol) added. The solution was allowed to warm to rt and stirred for a further 2H, after which DCM (100 mL) and H_2_O were added. The organic layer was washed with NaHCO_3_ (sat. aq.), then brine, dried (Na_2_SO_4_) and evaporated to dryness. The residue was purified by column chromatography on silica gel eluting with PE:EtOAc (1:0 → 0:1 v/v), where both solvents contained 0.5% v/v pyridine, to afford the *title compound* (736 mg, 60%) as a colourless glass; *R*_f_ = 0.67 (PE:EtOAc 1:3 v/v); ^1^H-NMR (400 MHz, CDCl_3_) δ 7.59 (dd, 2H, *J* = 8.0, 1.4), 7.57 (s, 1H, H-2), 7.51 (dd, 2H, *J* = 8.0, 1.3), 7.41-7.31 (m, 4H), 7.22 (t, 2H, *J* = 7.7), 6.13 (d, 1H, *J* = 2.1, H-1′), 5.75 (d, 1H, *J* = 1.6, H-1′′), 5.46 (dd, 1H, *J* = 6.4, 2.1, H-2′), 5.03 (dd, 1H, *J* = 6.4, 3.9, H-3′), 4.99 (dd, 1H, *J* = 6.2, 1.6, H-2′′), 4.96 (dd, 1H, *J* = 6.2, 3.8, H-3′′), 4.43 (ddd, 1H, *J* = 6.4, 5.8, 3.8, H-4′′), 4.34 (ddd, 1H, *J* = 6.0, 5.8, 3.9, H-4′), 4.24-4.11 (m, 2H, H-5″a and H-5″b), 3.80 (dd, 1H, *J* = 10.8, 5.6, H-5′a), 3.74 (dd, 1H, *J* = 10.8, 6.4, H-5′b), 1.61 (s, 3H, CH_3_), 1.60 (s, 3H, CH_3_), 1.428 (s, 9H, ^*t*^Bu), 1.427 (s, 9H, ^*t*^Bu), 1.37 (s, 3H, CH_3_), 1.36 (s, 3H, CH_3_), 1.00 (s, 9H, ^*t*^Bu) ppm; ^13^C-NMR (100 MHz, CDCl_3_) δ 154.5, 147.9, 145.6, 135.5 (2C), 135.4 (2C), 133.5, 132.8, 129.7, 129.6, 127.6 (2C), 127.4 (2C), 126.4, 125.3, 114.4, 114.2, 95.0, 91.2, 87.9, 87.2 (d, *J* = 7.8), 85.0, 83.3, 82.6 (d, 2C, *J* = 7.2), 81.6 81.5, 66.4 (d, *J* = 6.2), 63.9, 29.8 (3C), 29.7 (3C), 27.2, 27.1, 26.7 (3C), 25.5, 25.2, 19.2 ppm; ^31^P-NMR (162 MHz, ^1^H decoupled, CDCl_3_) δ -10.2 ppm; HRMS (ESI^+^) found *m/z* [M + Na]^+^ 1011.2911 and 1013.2900; C_45_H_62_N_4_O_12_^79^BrSiNaP requires 1011.2947, C_45_H_62_N_4_O_12_^81^BrSiNaP requires 1013.2926.

### *N*1-[2′′,3′′-*O*-isopropylidene-5′′-*O*-(di-*tert-*butyl)-phosphoryl-β-L-ribofuranosyl]-2′,3′-*O*-isopropylidene-8-bromoinosine (13)

Acetic acid (133 µL, 2.32 mmol) and TBAF.3H_2_O (698 mg, 2.21 mmol) were stirred in DMF (2 mL) for 30 min, after which the solution was cooled to 0 °C and **12** (730 mg, 0.74 mmol) in DMF (5 mL) added. The resulting solution was allowed to warm to rt and stirred for a further 4 h. The solution was diluted with ether, and NaHCO_3_ (sat. aq.) and NH_4_Cl (sat. aq.) added. The organic layer was separated, and the aqueous layer extracted with ether (×3). The combined organic layers were dried (Na_2_SO_4_), evaporated to dryness and the residue purified by column chromatography on silica gel eluting with DCM:Acetone (1:0 → 0:1 v/v), where both solvents contained 0.5% v/v pyridine, to afford the *title compound* (530 mg, 96%) as a colourless glass; *R*_f_ = 0.70 (DCM:Acetone 1:1 v/v); ^1^H-NMR (400 MHz, CDCl_3_) δ 8.10 (s, 1H, H-2), 6.06 (d, 1H, *J* = 4.7, H-1′), 5.96 (d, 1H, *J* = 1.8, H-1′′), 5.20 (dd, 1H, *J* = 5.8, 4.7, H-2′), 5.06-5.02 (m, 2H, H-2′′ and H-3′), 4.96 (dd, 1H, *J* = 6.4, 3.8, H-3′′), 4.44-4.41 (m, 2H, H-4′ and H-4′′), 4.24-4.12 (m, 2H, H-5′′a and H-5′′b), 3.87 (dd, 1H, *J* = 12.5, 2.5, H-5′a), 3.73 (dd, 1H, *J* = 12.5, 2.5, H-5′b), 1.64 (s, 3H, CH_3_), 1.56 (s, 3H, CH_3_), 1.45 (s, 9H, ^*t*^Bu), 1.43 (s, 9H, ^*t*^Bu), 1.36 (s, 3H, CH_3_), 1.33 (s, 3H, CH_3_) ppm; ^13^C-NMR (100 MHz, CDCl_3_) δ 154.3, 147.6, 146.1, 126.1, 125.8, 114.40, 114.38, 94.5, 93.0, 86.9 (d, *J* = 7.9), 85.8, 85.0, 82.9, 82.8 (d, *J* = 7.2), 82.7 (d, *J* = 7.2), 81.3, 81.2, 66.3 (d, *J* = 6.2), 62.8, 29.74 (d, 3C, *J* = 4.2), 29.72 (d, 3C, *J* = 4.2), 27.5, 27.1, 25.3, 25.2 ppm; ^31^P-NMR (162 MHz,^1^H decoupled, CDCl_3_) δ -10.4 ppm; HRMS (ESI^+^) found *m/z* [M + Na]^+^ 773.1744 and 775.1711; C_29_H_44_N_4_O_12_^79^BrNaP requires 773.1769, C_29_H_44_N_4_O_12_^81^BrNaP requires 775.1748.

### *N*1-[2′′,3′′-*O*-isopropylidene-5′′-*O*-(di-*tert-*butyl)phosphoryl-β-L-ribofuranosyl]-5′-*O*-[(diphenylthio)phosphoryl]- 2′,3′-*O*-isopropylidene-8-bromoinosine (14)

Intermediate **13** (250 mg, 0.33 mmol) was evaporated from pyridine (3 × 2 mL) and taken up in pyridine (2.5 mL). This solution was added to PSS (381 mg, 1.00 mmol) which had also been evaporated from pyridine (3 × 2 mL). 5-Phenyl-1*H*-tetrazole (146 mg, 1.00 mmol) and TPS-Cl (201 mg, 0.67 mmol) were added and the solution stirred at rt for 5H. DCM and H_2_O were added, the organic layer separated and the aqueous layer washed with DCM (×2). The combined organic layer was washed with brine, dried (Na_2_SO_4_) and evaporated to dryness. The residue was purified by column chromatography on silica gel eluting with PE:EtOAc (1:0 → 0:1 v/v), where both solvents contained 0.5% v/v pyridine, to afford the *title compound* (263 mg, 78%) as a white foam; *R*_f_ = 0.76 (DCM:Acetone 1:1 v/v); ^1^H-NMR (400 MHz, CDCl_3_) δ 7.97 (s, 1H, H-2), 7.47-7.25 (m, 10H, Ar-H), 6.17 (d, 1H, *J* = 2.0, H-1′), 5.79 (d, 1H, *J* = 1.5, H-1′′), 5.51 (dd, 1H, *J* = 6.2, 2.0, H-2′), 5.08 (dd, 1H, *J* = 6.2, 3.6, H-3′), 5.04 (dd, 1H, *J* = 6.4, 1.5, H-2′′), 4.97 (dd, 1H, *J* = 6.4, 3.9, H-3′′), 4.44-4.31 (m, 4H, H-4′, H-4′′, both H-5′), 4.24-4.21 (m, 2H, both H-5′′), 1.62 (s, 3H, CH_3_), 1.55 (s, 3H, CH_3_), 1.47 (s, 9H, ^*t*^Bu), 1.45 (s, 9H, ^*t*^Bu), 1.39 (s, 3H, CH_3_), 1.30 (s, 3H, CH_3_) ppm; ^13^C-NMR (100 MHz, CDCl_3_) δ 154.5, 148.0, 146.4, 135.3 (d, 2C, *J* = 5.5), 135.1 (d, 2C, *J* = 5.5), 129.6-129.5 (m, 2C), 129.4-129.3 (m, 4C), 126.3, 125.6-125.5 (m, 2C), 125.4, 114.8, 114.2, 95.5, 91.2, 87.5 (d, *J* = 7.8), 85.5 (d, *J* = 8.4), 84.8, 83.5, 83.1 (d, *J* = 7.4), 83.0 (d, *J* = 7.4), 81.7, 81.1, 66.8 (d, *J* = 6.0), 66.2 (d, *J* = 8.2), 29.8 (d, 3C, *J* = 4), 29.7 (d, 3C, *J* = 4), 27.2, 27.0, 25.4, 25.2 ppm; ^31^P-NMR (162 MHz,^1^H decoupled, CDCl_3_) δ 50.7, −10.8 ppm; HRMS (ESI^+^) found *m/z* [M + Na]^+^ 1037.1639 and 1039.1569; C_41_H_53_N_4_O_13_^79^BrNaP_2_S_2_ requires 1037.1601, C_41_H_53_N_4_O_13_^81^BrNaP_2_S_2_ requires 1039.1581.

### *N*1-[5′′-*O*-phosphoryl-β-L-ribofuranosyl]-5′-*O*-[(diphenylthio)phosphoryl]-8-bromoinosine (15)

Intermediate **14** (250 mg, 0.25 mmol) was stirred in 50% aq. TFA (4 mL) at 0 °C for 4H. All solvents were evaporated and the residue co-evaporated with MeOH (×4). The residue was purified by column chromatography on silica gel eluting with EtOAc:MeOH:H_2_O (1:0:0 → 4:2:0 → 7:2:1 v/v/v) to afford the *title compound* (147 mg, 70%) as a colourless glass; *R*_f_ = 0.14 (EtOAc:MeOH:H_2_O 7:2:1 v/v/v); ^1^H-NMR (500 MHz, d_4_-MeOD) δ 8.78 (s, 1H, H-2), 7.44-7.37 (m, 6H), 7.32-7.28 (m, 4H) (10 × Ar-H), 6.15 (d, 1H, *J* = 3.9, H-1′), 6.01 (d, 1H, *J* = 3.3, H-1′′), 5.22 (dd, 1H, *J* = 5.4, 3.3, H-2′′), 4.86 (t, 1H, *J* = 5.9, H-3′′), 4.50-4.43 (m, 2H, both H-5′′), 4.32 (dd, 1H, *J* = 4.7, 3.9, H-3′), 4.28 (dd, 1H, *J* = 4.7, 3.9, H-2'), 4.21-4.10 (m, 4H, H-4′, H-4′′ and both H-5′) ppm; ^13^C-NMR (125 MHz, d_4_-MeOD) δ 157.2, 150.0, 146.9, 136.6 (d, 2C, *J* = 5.3), 136.4 (d, 2C, *J* = 5.4), 131.0 (d, *J* = 3.1), 130.9 (d, *J* = 3.1), 130.5 (4C), 128.7, 126.8 (d, *J* = 6.4), 126.7 (d, *J* = 6.4), 125.4, 93.3, 90.6, 85.5 (d, *J* = 8.6), 83.9 (d, *J* = 8.0), 77.3, 73.7, 71.1, 70.9, 68.3 (d, *J* = 8.3), 64.9 (d, *J* = 5.0) ppm; ^31^P-NMR (202 MHz, ^1^H decoupled, d_4_-MeOD) δ 52.1, 0.7 ppm; HRMS (ESI^−^) found *m/z* [M − H]^−^ 820.9762 and 822.9735; C_27_H_28_N_4_O_13_^79^BrP_2_S_2_ requires 820.9758, C_27_H_28_N_4_O_13_^81^BrP_2_S_2_ requires 822.9738.

### *N*1-(5′′-*O*-phosphoryl-β-L-ribofuranosyl)-5′-*O*-(phenylthio)phosphoryl-8-bromoinosine (16)

Intermediate **15** (24 mg, 0.029 mmol) was taken up in dioxane:H_2_O (1 mL, 1:1 v/v). NaOH (100 µL, 1 M) was added and the solution stirred for 30 min at rt before addition of HCl (100 µL, 1 M). The solution was diluted with H_2_O and washed with hexane (×3) before evaporation of all solvents to give a colourless glass which was converted to the TEA salt as described below. ^1^H-NMR (500 MHz, D_2_O) δ 8.40 (s, 1H, H-2), 7.01 (d, 2H, *J* = 7.9), 6.92 (t, 1H, *J* = 7.5), 6.80 (t, 2H, *J* = 7.7) (5 × Ar-H), 5.95 (d, 1H, *J* = 6.0, H-1′), 5.78 (d, 1H, *J* = 2.8, H-1″), 5.54 (t, 1H, *J* = 6.0, H-2′), 4.43 (dd, 1H, *J* = 6.0, 2.8, H-3′), 4.29-4.24 (m, 2H), 4.21-4.17 (m, 4H), 4.08-4.01 (m, 2H) ppm; ^13^C-NMR (125 MHz, D_2_O) δ 156.0, 148.8, 145.0, 131.5 (d, 2C, *J* = 5.5), 129.7 (d, *J* = 5.6), 128.8, 128.4 (2C), 127.2, 123.7, 90.4, 89.9, 84.5 (d, *J* = 10.6), 82.8 (d, *J* = 8.8), 75.0, 70.8, 70.3, 68.8, 65.8 (d, *J* = 5.4), 63.9 (d, *J* = 4.8) ppm; ^31^P-NMR (202 MHz, D_2_O, ^1^H-decoupled) δ 17.3, 0.0 ppm; HRMS (ESI^−^) calcd for C_21_H_23_N_4_O_14_P_2_S^79^BrNa 750.9493 [(M + Na − 2H)^−^], found 750.9493; and calcd for C_21_H_23_N_4_O_14_P_2_S^81^BrNa 752.9473 [(M + Na − 2H)^−^], found 752.9458. Conversion to TEA salt: The Na^+^ salt was passed through pre-washed DOWEX H^+^ resin. Acidic fractions were neutralised with TEAB (2 mL, 1 M). All solvents were evaporated and the residue co-evaporated with H_2_O to remove excess buffer. The colorless glass obtained was used directly for cyclisation.

### Cyclic-8-bromoinosine 5′-diphosphate-L-ribose (8-Br-L-cIDPR, 6)

Intermediate **16** (0.029 mmol) was evaporated from pyridine (2 mL, ×3). The residue was taken up in pyridine (20 mL) and added over 15H to a solution of iodine (140 mg, 0.591 mmol) and 3 Å molecular sieves (1.0 g) in pyridine (40 mL), in the dark. The solution was filtered through celite, washed with H_2_O. After addition of TEAB (2 mL) all solvents were evaporated, and the residue partitioned between H_2_O and CHCl_3_. The aqueous layer was washed with CHCl_3_ and evaporated to dryness. The residue was purified by semi-preparative reverse phase HPLC eluted at 5 mL/min with acetonitrile:0.1 M TEAB (1:19 → 13:7 v/v) over 25 min. Fractions were analysed by analytical RP-HPLC and appropriate fractions collected and evaporated under vacuum to give the *title compound* (3.2 mg, 18% over 2 steps); UV (H_2_O, pH 7), λ_max_ 255 nm (ε 12,400); ^1^H-NMR (500 MHz, D_2_O) δ 8.80 (s, 1H, H-2), 6.34 (d, 1H, *J* = 3.8, H-1″), 6.05 (d, 1H, *J* = 6.8, H-1′), 5.15 (dd, 1H, *J* = 6.8, 5.0, H-2′), 4.61 (dt, 1H, *J* = 10.6, 1.9, H-5″a), 4.54 (t, 1H, *J* = 5.0, H-3″), 4.51 (dd, 1H, *J* = 5.0, 3.8, H-2″), 4.47 (dd, 1H, *J* = 5.0, 1.8, H-3′), 4.35 (dd, 1H, *J* = 6.4, 1.8, H-4′), 4.27-4.22 (m, 2H, H-4″ and H-5′a), 4.09-4.02 (m, 2H, H-5′b and H-5″b) ppm; ^13^C-NMR (125 MHz, D_2_O) δ 156.8, 148.8, 145.3, 128.1, 123.7, 91.0, 87.9, 85.2 (d, *J* = 10.6), 83.6 (d, *J* = 9.0), 75.1, 73.3, 70.9, 69.5, 64.9 (d, *J* = 4.8), 64.3 (d, *J* = 5.0) ppm; ^31^P-NMR (202 MHz, D_2_O, ^1^H-decoupled) δ -10.39 (d, *J* = 14.1), −10.71 (d, *J* = 14.1) ppm; HRMS (ESI^−^) calcd for C_15_H_18_N_4_O_14_P_2_^79^Br 618.9484 [(M − H)^−^], found 618.9493; and calcd for C_15_H_18_N_4_O_14_P_2_^81^Br 620.9463 [(M−H)^−^], found 620.9464.

### Cyclic-inosine 5′-diphosphate-L-ribose (L-cIDPR, 5)

Cyclic-8-bromoinosine 5′-diphosphate-L-ribose (**6**, 10 mg, 0.016 mmol) was taken up in MilliQ H_2_O (4 mL) and NaHCO_3_ (13 mg, 0.161 mmol) added. EtOH (2 mL) and Pd/C (5 mg) were added and the flask placed under an atmosphere of H_2_ for 16H. The solution was filtered and all solvents were evaporated. The residue was purified by semi-preparative reverse phase HPLC eluted at 5 mL/min with acetonitrile:0.1 M TEAB (1:19 → 13:7 v/v) over 25 min. Fractions were analysed by analytical RP-HPLC and appropriate fractions collected and evaporated under vacuum to give the *title compound* (4.0 mg, 46% over 2 steps); UV (H_2_O, pH 7), λ_max_ 251 nm (ε 9,900); ^1^H-NMR (500 MHz, D_2_O) δ 8.81 (s, 1H, 2H-2), 8.11 (s, 1H, H-8), 6.35 (d, 1H, *J* = 3.5, H-1″), 5.93 (d, 1H, *J* = 6.5, H-1′), 5.09 (dd, 1H, *J* = 6.5, 4.9, H-2′), 4.59-4.52 (m, 3H, H-5″a, H-3″, H-2″), 4.49 (dd, 1H, *J* = 4.9, 1.9, H-3′), 4.35-4.33 (m, 1H, H-4′), 4.29-4.27 (m, 1H, H-4″), 4.23 (dd, 1H, *J* = 11.7, 3.5, H-5′a), 4.09-4.04 (m, 2H, H-5′b and H-5″b) ppm; ^13^C-NMR (125 MHz, D_2_O) δ 158.0, 147.6, 145.1, 142.4, 123.7, 90.5, 88.1, 84.9 (d, *J* = 11.1), 83.7 (d, *J* = 9.3), 75.1, 73.4, 71.0, 69.6, 65.0 (d, *J* = 5.5), 64.2 (d, *J* = 4.6) ppm; ^31^P-NMR (202 MHz, D_2_O, ^1^H-decoupled) δ −10.40 (d, *J* = 10.1), −10.75 (d, *J* = 10.1) ppm; HRMS (ESI^−^) calcd for C_15_H_19_N_4_O_14_P_2_ 541.0378 [(M − H)^−^], found 541.0390.

### Cyclic-8-aminoinosine 5′-diphosphate-L-ribose (8-NH_2_-L-cIDPR, 7)

Cyclic-8-bromoinosine 5′-diphosphate-L-ribose (**6**, 3.6 mg, 5.8 μmol) was converted to the H^+^ form by stirring with DOWEX 50WX8H^+^ resin in MilliQ (2 mL) for 15 mins. The resin was removed by filtration and all solvent evaporated. The residue was co-evaporated with DMF (4 × 1 mL) and taken up in DMF (1 mL). TMSN_3_ (50 μL) was added and the solution stirred at 70 °C in the dark for 16H at which point the HPLC showed 95% conversion to the 8-azido product. MilliQ (5 mL) was added and all solvents evaporated. The residue was taken up in TEAB (0.05 M, 5 mL) and dithiothreitol (10 mg) added. The resulting solution was stirred for 12H, when HPLC analysis showed complete consumption of the 8-azido intermediate. The solution was purified by RP-HPLC eluted at 5 mL/min with acetonitrile:0.1 M TEAB (1:19 → 13:7 v/v) over 25 min. Fractions were analysed by analytical RP-and appropriate fractions collected and evaporated under vacuum to give the *title compound* (1.35 μmol, 23% over 2 steps); UV (H_2_O, pH 7), λ_max_ 261 nm (ε 11,700); ^1^H-NMR (500 MHz, D_2_O) δ 8.60 (s, 1H, H-2), 6.37 (d, 1H, *J* = 3.5, H-1″), 5.80 (d, 1H, *J* = 6.7, H-1′), 5.20 (dd, 1H, *J* = 6.7, 4.9, H-2′), 4.56-4.53 (m, 3H, H-5″a, H-3″, H-2″), 4.48 (dd, 1H, *J* = 4.9, 1.8, H-3′), 4.30 (dd, 1H, *J* = 6.2, 1.8, H-4′), 4.27-4.25 (m, 1H, H-4″), 4.20-4.18 (m, 1H, H-5′a), 4.06 (d, 1H, H-5′b), 4.02 (dt, 1H, *J* = 10.8, 4.0, H-5″b) ppm; ^13^C-NMR (125 MHz, D_2_O) δ 152.6, 149.9, 145.9, 145.1, 110.5, 88.6, 88.3, 85.1 (d, *J* = 10.8), 83.4 (d, *J* = 8.6), 74.8, 72.9, 70.5, 69.2, 64.9 (d, *J* = 3.8), 64.2 (d, *J* = 4.3) ppm; ^31^P-NMR (202 MHz, D_2_O, ^1^H-decoupled) δ −10.27 (d, *J* = 16.2), −10.51 (d, *J* = 16.2) ppm; HRMS (ESI^−^) calcd for C_15_H_20_N_5_O_14_P_2_ 556.0487 [(M − H)^−^], found 556.0471.

#### Enzymatic Assay for cADPR Hydrolysis

The inhibition of cADPR hydrolysis by various concentrations of analogue (0–1 mM) was determined by incubating 1 μM cADPR with 1 μg/ml of CD38 for 10 min at 20–24 °C in 25 mM sodium acetate, pH 4.5. The reaction was stopped by the addition of 150 mM HCl. The precipitated protein was filtered, and the pH was neutralised with Tris base. After diluting the mixture 20-fold, the concentration of the unhydrolysed cADPR present in the diluted reaction mixture was assayed by the fluorimetric cycling assay as described previously^42^.

#### Modelling of the L-ribose compounds: Method A

Three cIDPR analogues with L-ribose in the “northern” position but differing in the substituent at the 8-position (hydrogen, bromine or amino) were built using the Schrödinger software and docked into the 2PGJ structure using GOLD^50^. The binding site was defined as a sphere of 5 Å radius centred on the centroid of the cIDPR: the centroid of the docked ligand has to lie within this sphere. Each ligand was docked twenty-five times. The best ranked poses of each the ligand were merged with the protein structure and the resulting complexes passed through a minimisation procedure using the Schrödinger software.

#### Method B

The ligand in the prepared 2PGJ and 3U4H crystal structures was modified so that the “northern” ribose was of the L-configuration. An 8-amino or 8-bromo group was added to the 8-position of the 2PGJ ligand, and the 8-amino group of the 3U4H ligand was either amended to an 8-bromo substituent or a hydrogen. The resulting complexes were put through 500 rounds of minimisation using the Schrödinger software or minimised using the Discovery Studio software from Accelrys. All figures were prepared using PyMOL.

#### HPLC studies

The solution containing the CD38 catalytic domain was adjusted to the desired concentration using Tris-HCl buffer (20 mM, pH 8) and 50 μL therefrom was added to the inhibitor (0.05 μmole) in an Eppendorf tube at room temperature. At a given time point, a sample of 5 μL was removed and diluted with 95 μL MilliQ water. 10 μL Of this sample was injected directly into the analytical HPLC system (see General Experimental), eluting at 1 mL/min with an isocratic ion-pair buffer: 0.17% (m/v) cetrimide and 45% (v/v) phosphate buffer (pH 6.4) in MeOH.

### Data availability

The crystallographic data for 2PGJ and 3U4H are available in the PDB repository (www.pcsb.org). All other data generated or analysed during this study are included in this published article and Supplementary Information file.

## Electronic supplementary material

Supplementary information
