# Supplementary material for: Second messenger analogues highlight unexpected substrate sensitivity of CD38: total synthesis of the hybrid “L-cyclic inosine 5′-diphosphate ribose”
Source: Sci Rep. 2017 Nov 23;7:16100. doi: 10.1038/s41598-017-16388-0 (PMC5700923; doi:10.1038/s41598-017-16388-0)
Supplement: Supplementary file 1 — Supplementary information [file 41598_2017_16388_MOESM1_ESM.pdf]

Supplementary Information

Second messenger analogues highlight unexpected substrate sensitivity of CD38: total synthesis of the hybrid "L-cyclic inosine 5'-diphosphate ribose"

Joanna M. Watt[1,3], Richard Graeff,[2] Mark P. Thomas[1] and Barry V. L. Potter[1,3]*.

1. Wolfson Laboratory of Medicinal Chemistry, Department of Pharmacy and Pharmacology, University of Bath, Claverton Down, Bath, BA2 7AY, UK
2. Department of Physiology, University of Hong Kong, Hong Kong, China
3. Medicinal Chemistry and Drug Discovery, Department of Pharmacology, University of Oxford, Mansfield Road, Oxford, OX1 3QT, UK

Contents: **Page**

Supplementary Figure S1: HPLC studies of cIDPR and novel analogues with shCD38 2

Supplementary Figure S2: Crystal structure data showing *Aplysia* cyclase bound to both *N*1-hydrolysed IDPR and cIDPR in an up-side-down orientation. 3

Supplementary Figure S3: Molecular modelling of 8-NH_2-L-cIDPR 4

^{1}H-NMR and ^{13}C-NMR of compounds **5-7** and **9-15**. 5

Supplementary Figure S1: HPLC studies of cIDPR and novel analogues with shCD38.

cIDPR with CD38 (1 ug per mL) T = 0 (have 2h, 5h) 24h

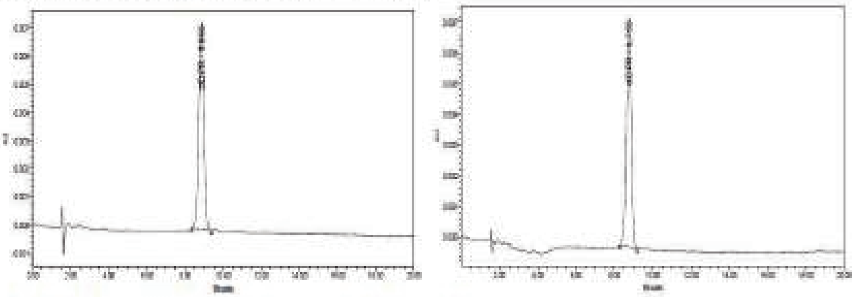

cIDPR with CD38 (4 mg per mL) T = 0 (have 1.5h) 7h, (22h)

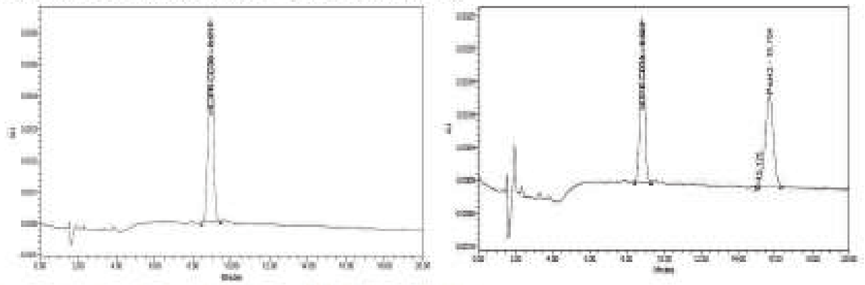

L-cIDPR with CD38 (4 mg per mL) T = 0 (have 1h, 3h) 6h (23h)

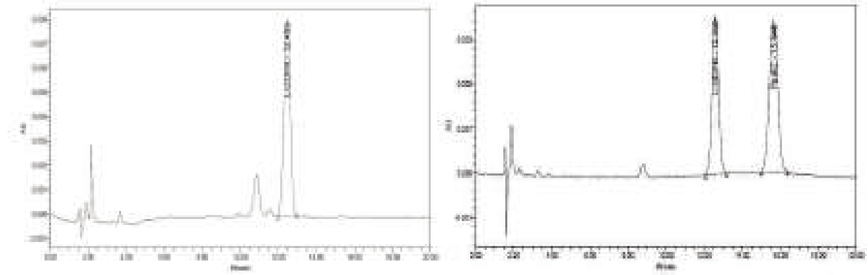

8-Br-L-cIDPR with CD38 (4 mg per mL) T = 0 (have 1h, 3h) 19h.

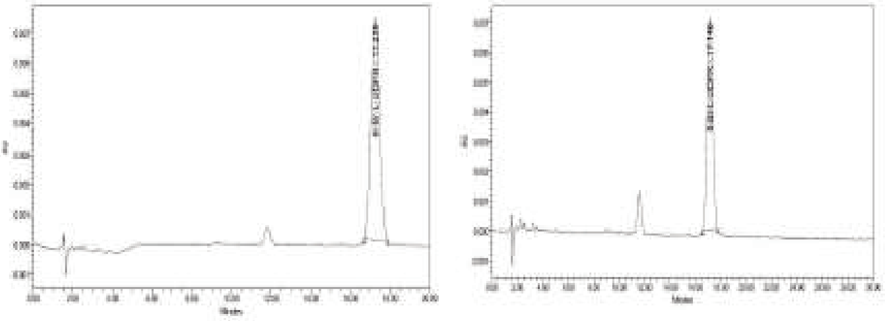

At a given time point, a sample of 5 µL was removed and diluted with 95 µL MilliQ water. 10 µL Of this sample was injected directly into the analytical HPLC. Traces are a result of elution at 1 mL/min with an isocratic ion-pair buffer: 0.17% (m/v) cetrimide and 45% (v/v) phosphate buffer (pH 6.4) in MeOH.

Supplementary Figure S2: Crystal structure data showing *Aplysia* cyclase bound to both *N*1-hydrolysed IDPR and cIDPR in an up-side-down orientation.

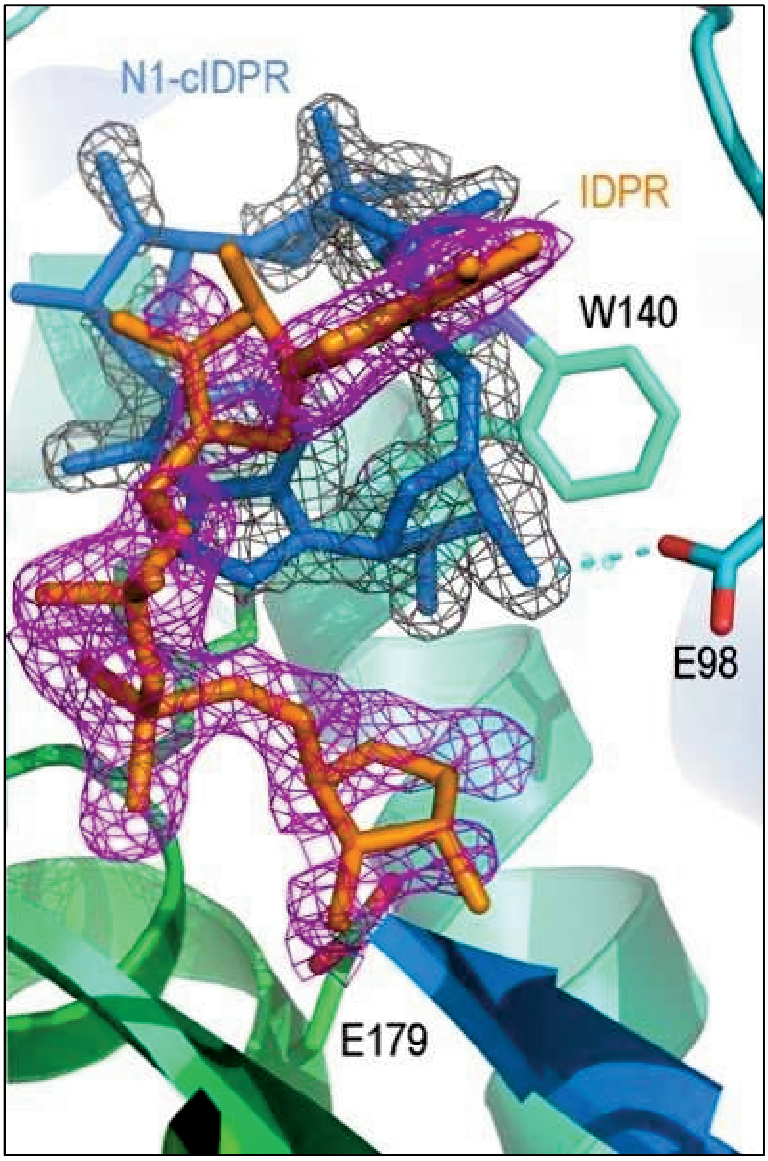

X-ray analysis of *N*1-cIDPR complexed with wild-type *Aplysia* cyclase, observed after crystal soaking with 30 nM *N*1-cIDPR. *N*1-cIDPR was found clearly to be partially hydrolyzed to IDPR at the *N*1 position (electron density shown in magenta, structure shown as orange sticks). *N*1-cIDPR is also present (electron density shown in grey, structure shown as blue sticks), in an up-side-down and left-side-right orientation compared with *N*1-cIDPR in CD38 (Q Liu, C Moreau, Q Hao, B V L Potter unpublished data).

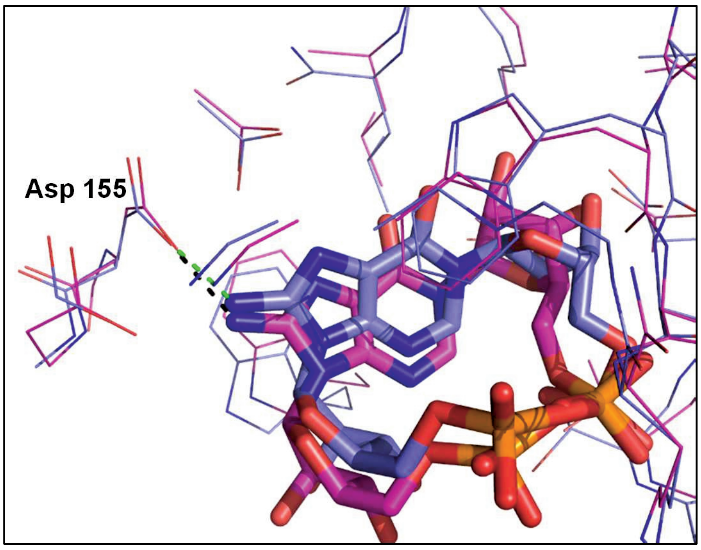

Supplementary Figure S3: 3U4H with 8-NH_2-cIDPR (pink) and 8-NH_2-L-cIDPR (purple) showing predicted H-bonds to Asp-155.

^{1}H-NMR, ^{13}C-NMR and ^{31}P-NMR for novel compounds

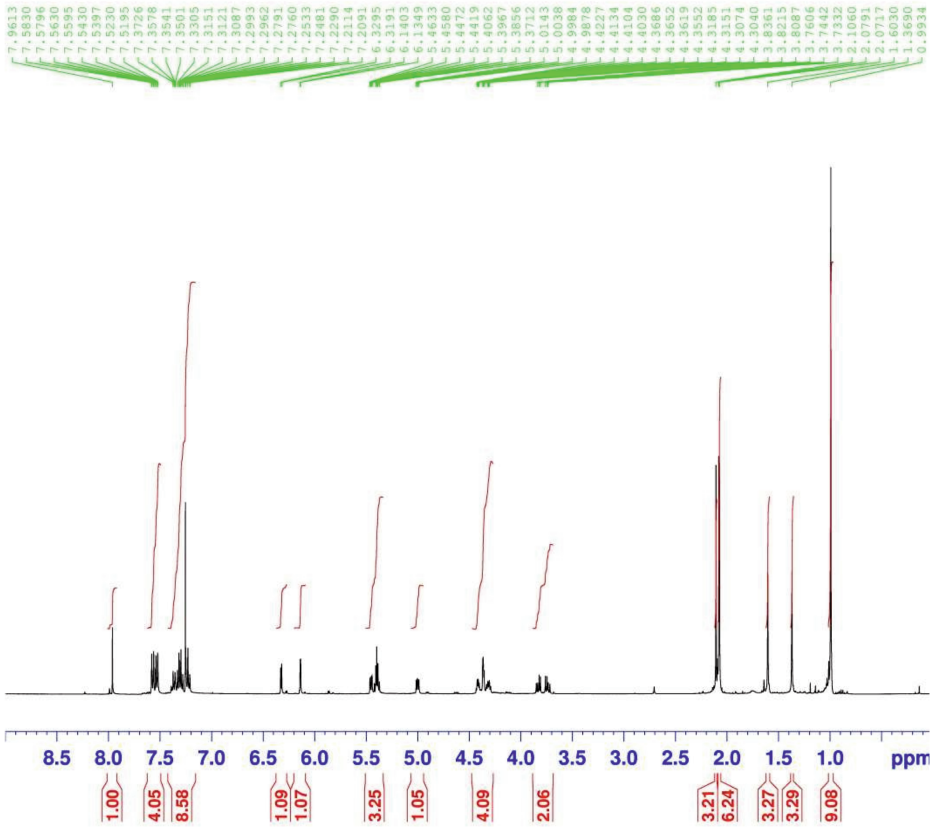

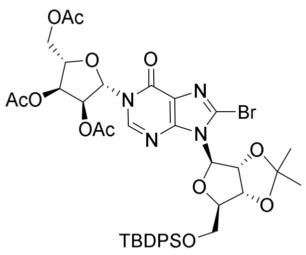

Compound **9**

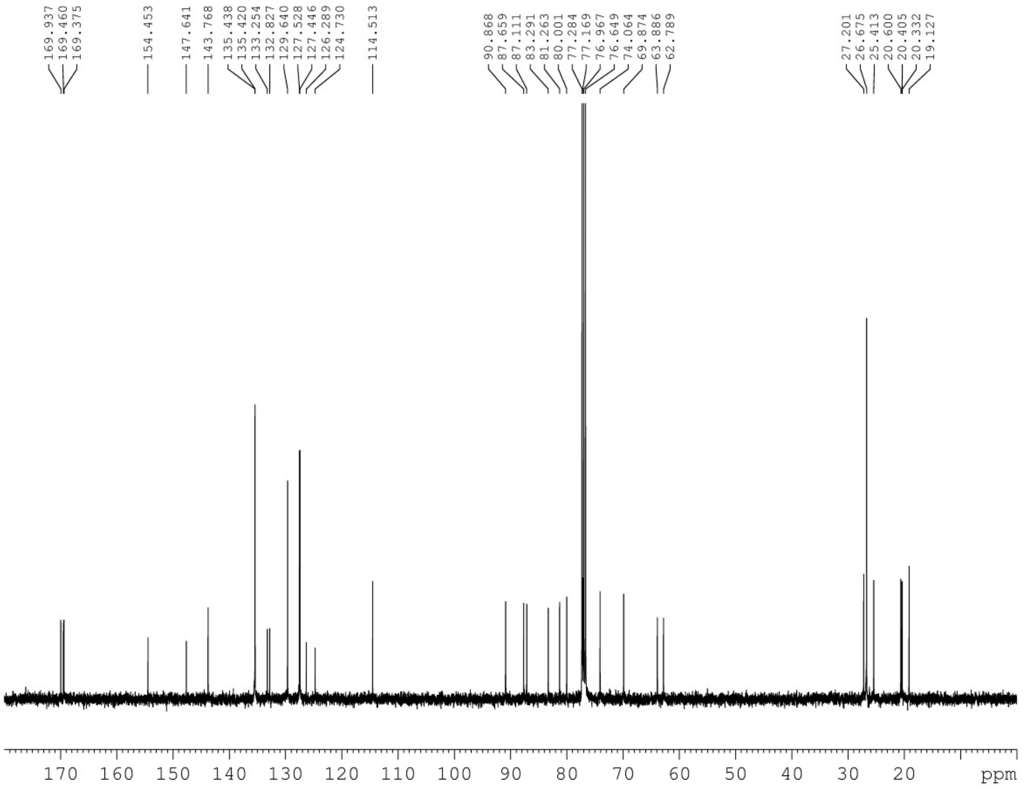


```
NAME        Feb08-2012-JMS16169
EXPNO                        11
PROCNO                        1
Date_                  20120209
Time                       1.47
INSTRUM                AVIII400
PROBHD       5 mm PABBO BB-
PULPROG                  zgpg30
TD                        65536
SOLVENT                   CDCl3
NS                          512
DS                            4
SWH                   24038.461 Hz
FIDRES                 0.366798 Hz
AQ                    1.3631988 sec
RG                         1820
DW                       20.800 usec
DE                         6.50 usec
TE                        298.0 K
D1                   2.00000000 sec
D11                  0.03000000 sec
TD0                           1

======== CHANNEL f1 ========
NUC1                        13C
P1                         8.75 usec
PL1                       -2.00 dB
PL1W                58.91986084 W
SFO1                100.6001970 MHz

======== CHANNEL f2 ========
CPDPRG2                 waltz16
NUC2                         1H
PCPD2                     80.00 usec
PL2                        0.00 dB
PL12                      15.78 dB
PL13                      19.00 dB
PL2W                 9.74611950 W
PL12W                0.25753233 W
PL13W                0.12269637 W
SFO2                400.0416002 MHz
SI                        65536
SF                  100.5901380 MHz
WDW                          EM
SSB                           0
LB                         1.00 Hz
GB                            0
PC                         1.40
```

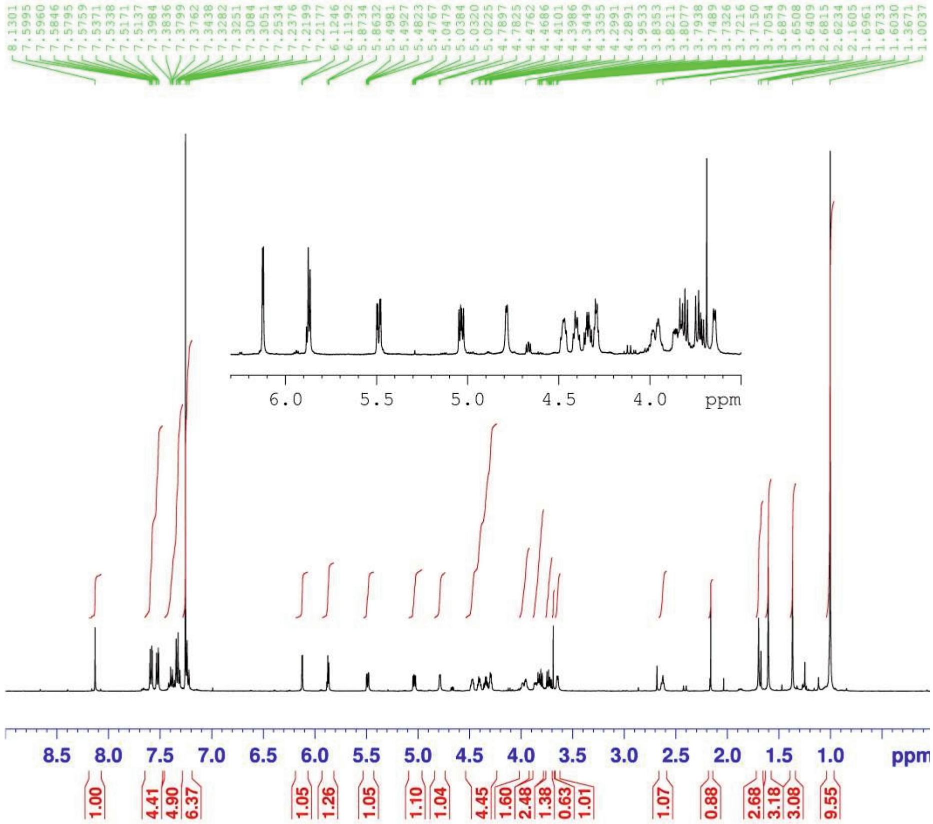

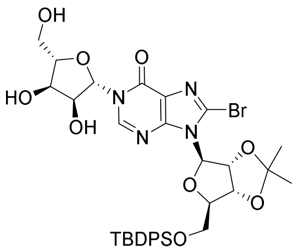

Compound **10**

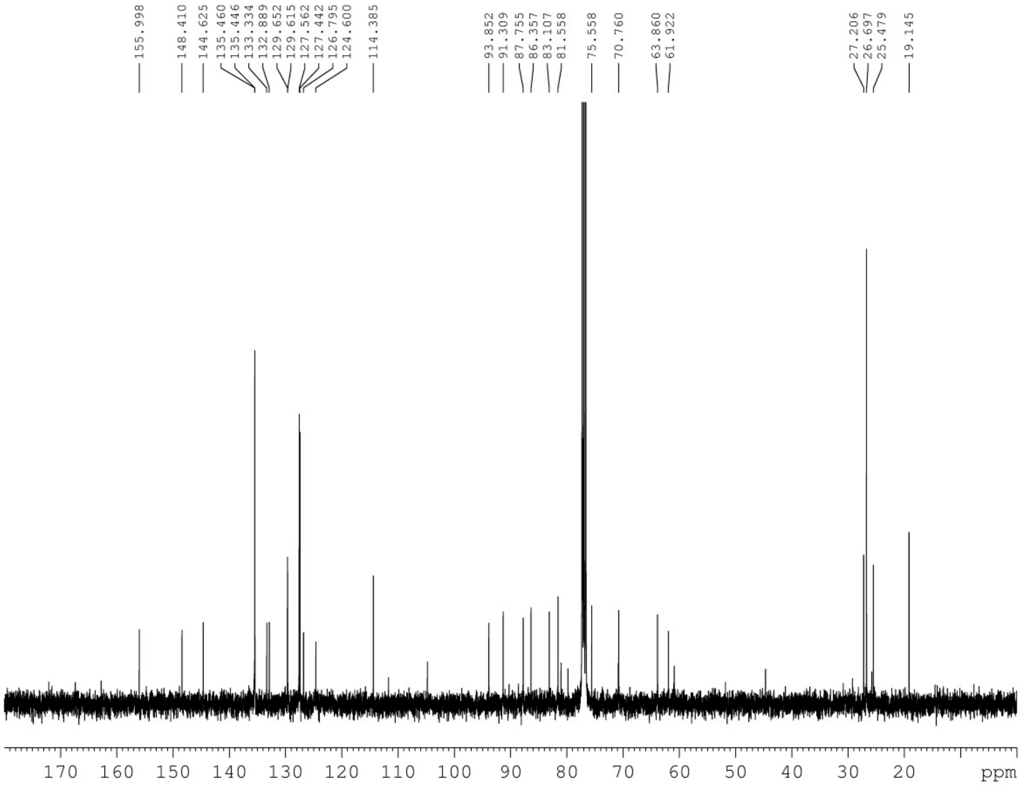

NAME	Nov07-2011-JMS18482	
EXPNO	14	
PROCNO	1	
Date_	20111108	
Time	3.47	
INSTRUM	AVIII400	
PROBHD	5 mm PABBO BB-	
PULPROG	zgpg30	
TD	65536	
SOLVENT	CDCl3	
NS	2048	
DS	4	
SWH	24038.461	Hz
FIDRES	0.366798	Hz
AQ	1.3631988	sec
RG	2050	
DW	20.800	usec
DE	6.50	usec
TE	298.0	K
D1	2.00000000	sec
D11	0.03000000	sec
TD0	1	
======== CHANNEL f1 ========		
NUC1	13C	
P1	8.75	usec
PL1	-2.00	dB
PL1W	58.91986084	W
SFO1	100.6001970	MHz
======== CHANNEL f2 ========		
CPDPRG2	waltz16	
NUC2	1H	
PCPD2	80.00	usec
PL2	0.00	dB
PL12	15.78	dB
PL13	19.00	dB
PL2W	9.74611950	W
PL12W	0.25753233	W
PL13W	0.12269637	W
SFO2	400.0416002	MHz
SI	65536	
SF	100.5901380	MHz
WDW	EM	
SSB	0	
LB	1.00	Hz
GB	0	
PC	1.40	

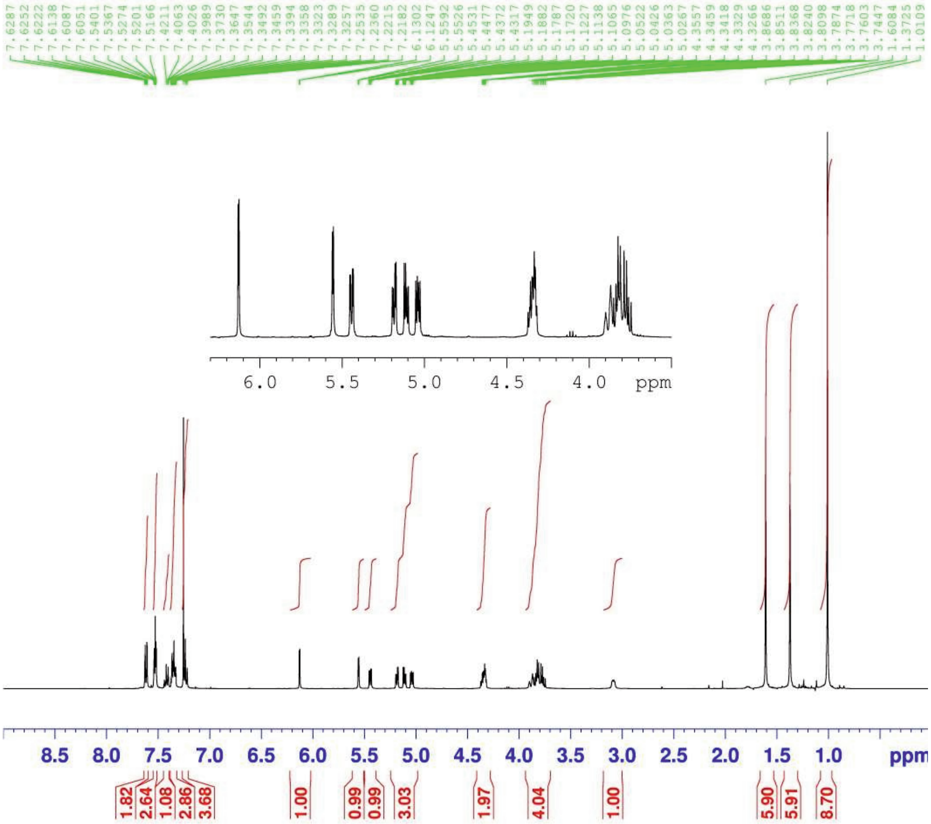

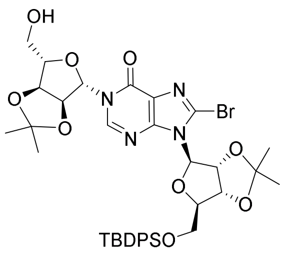

Compound **11**

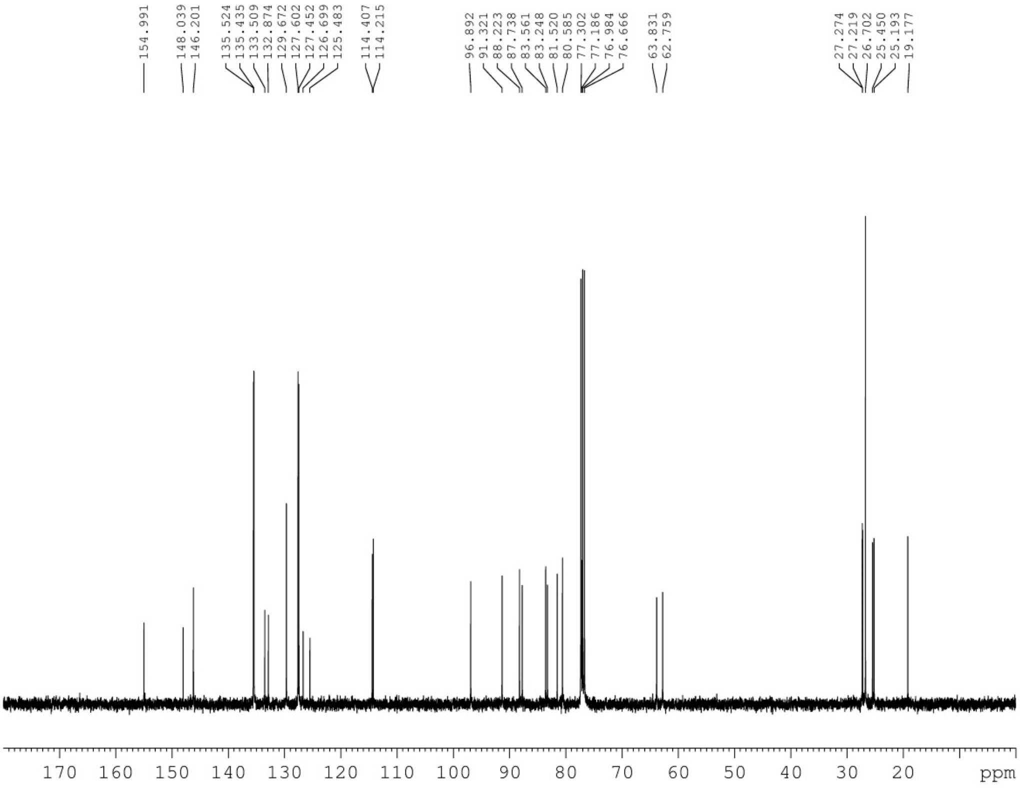


```
NAME        Nov08-2011-JMS18564
EXPNO                    11
PROCNO                    1
Date_              20111108
Time                  19.18
INSTRUM            AVIII400
PROBHD    5 mm PABBO BB-
PULPROG              zgpg30
TD                    65536
SOLVENT               CDCl3
NS                      256
DS                        4
SWH               24038.461 Hz
FIDRES             0.366798 Hz
AQ                1.3631988 sec
RG                     1820
DW                   20.800 usec
DE                     6.50 usec
TE                    298.0 K
D1               2.00000000 sec
D11              0.03000000 sec
TD0                       1

======== CHANNEL f1 ========
NUC1                    13C
P1                     8.75 usec
PL1                   -2.00 dB
PL1W            58.91986084 W
SFO1           100.6001970 MHz

======== CHANNEL f2 ========
CPDPRG2             waltz16
NUC2                     1H
PCPD2                 80.00 usec
PL2                    0.00 dB
PL12                  15.78 dB
PL13                  19.00 dB
PL2W             9.74611950 W
PL12W            0.25753233 W
PL13W            0.12269637 W
SFO2           400.0416002 MHz
SI                    65536
SF             100.5901380 MHz
WDW                      EM
SSB                       0
LB                     1.00 Hz
GB                        0
PC                     1.40
```

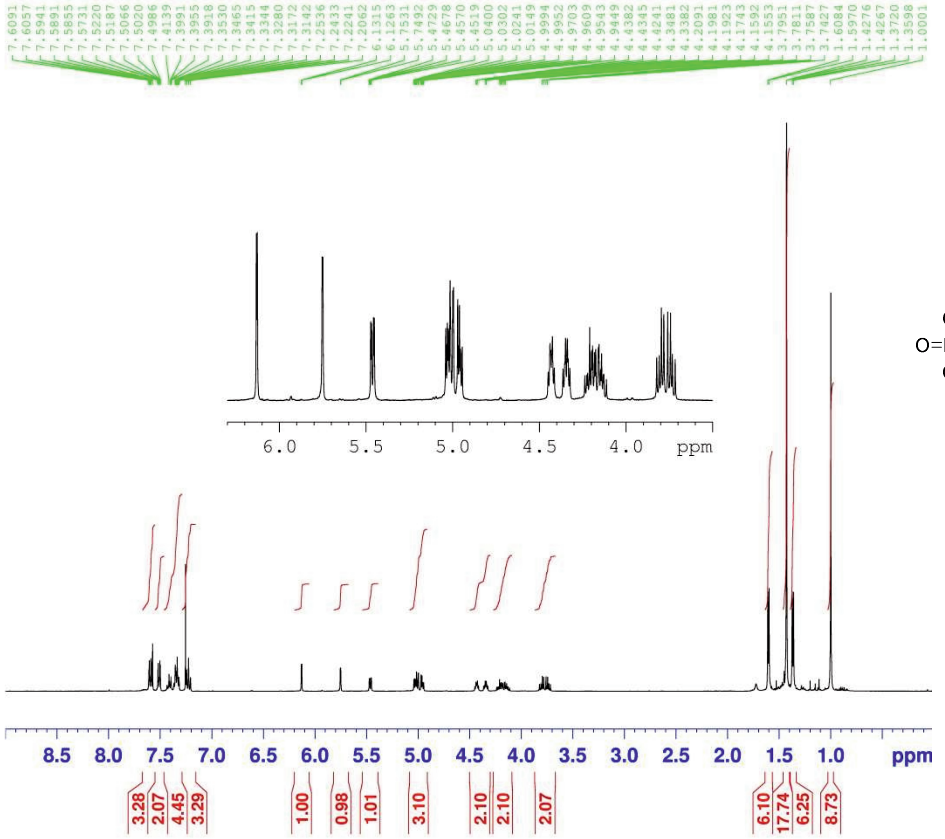
7.6091 7.6057 7.5941 7.5891 7.5855 7.5731 7.5220 7.5187 7.5066 7.5020 7.4986 7.4139 7.3991 7.3955 7.3918 7.3530 7.3465 7.3415 7.3344 7.3280 7.3172 7.3142 7.2536 7.2433 7.2241 7.2062 6.1315 6.1263 5.7531 5.7492 5.4729 5.4678 5.4570 5.4519 5.0400 5.0302 5.0241 5.0149 4.9994 4.9952 4.9703 4.9609 4.9543 4.9449 4.4382 4.4345 4.4241 4.3481 4.3382 4.2091 4.1981 4.1923 4.1743 4.1592 4.1553 3.7951 3.7811 3.7587 3.7427 1.6084 1.5970 1.5776 1.4267 1.3720 1.3598 1.0001
6.0 5.5 5.0 4.5 4.0 ppm
8.5 8.0 7.5 7.0 6.5 6.0 5.5 5.0 4.5 4.0 3.5 3.0 2.5 2.0 1.5 1.0 ppm
3.28 2.07 4.45 3.29 1.00 0.98 1.01 3.10 2.10 2.10 2.07 6.10 17.74 6.25 8.73

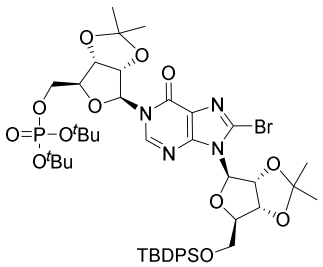
O
O
O
N
Br
O=P–O^{t}Bu
O^{t}Bu
N
N
N
O
O
O
TBDPSO

Compound **12**

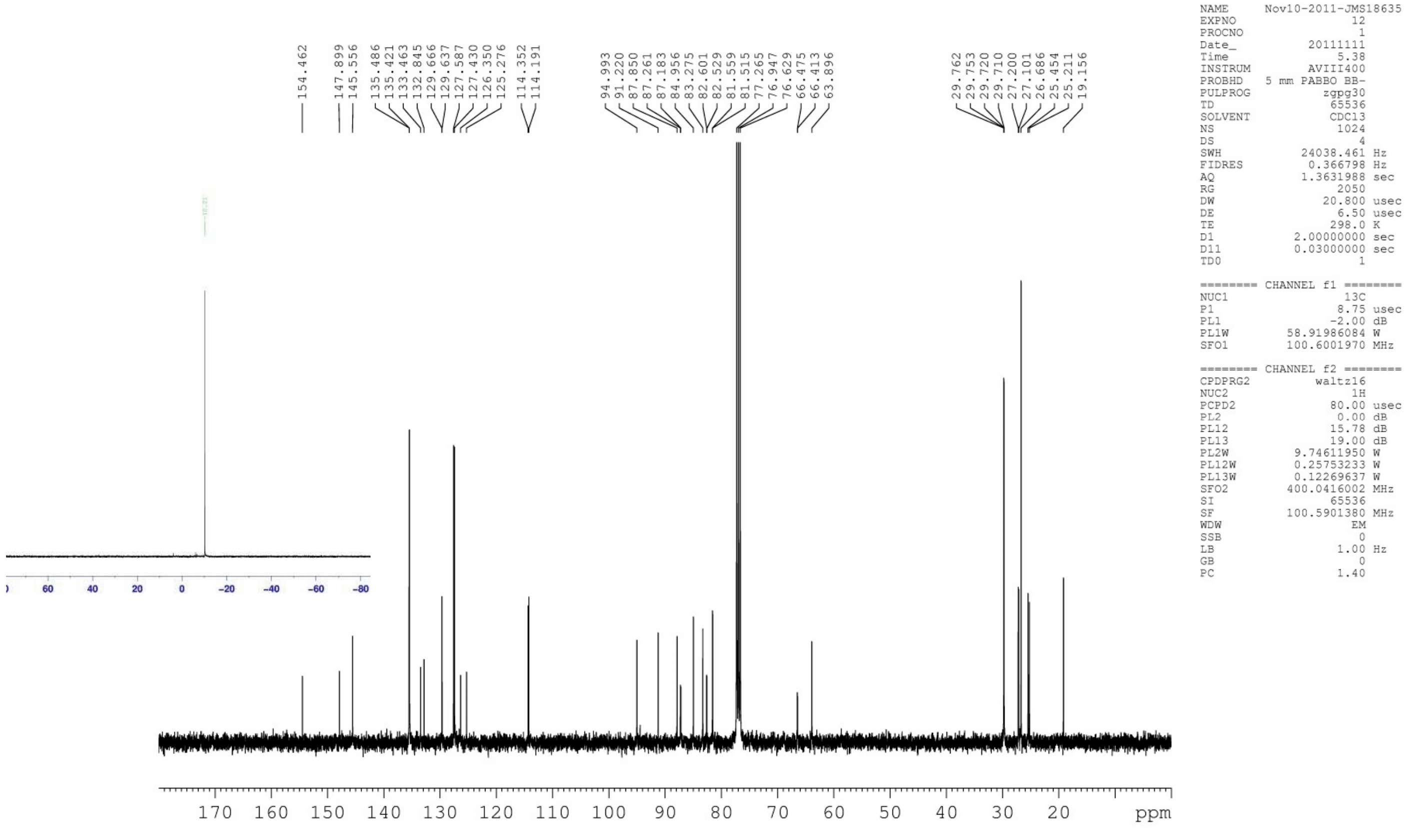
154.462 147.899 145.556 135.486 135.421 133.463 132.845 129.666 129.637 127.587 127.430 126.350 125.276 114.352 114.191
94.993 91.220 87.850 87.261 87.183 84.956 83.275 82.601 82.529 81.559 81.515 77.265 76.947 76.629 66.475 66.413 63.896
29.762 29.753 29.720 29.710 27.200 27.101 26.686 25.454 25.211 19.156
60 40 20 0 -20 -40 -60 -80
170 160 150 140 130 120 110 100 90 80 70 60 50 40 30 20 ppm
NAME Nov10-2011-JMS18635
EXPNO 12
PROCNO 1
Date_ 20111111
Time 5.38
INSTRUM AVIII400
PROBHD 5 mm PABBO BB-
PULPROG zgpg30
TD 65536
SOLVENT CDCl3
NS 1024
DS 4
SWH 24038.461 Hz
FIDRES 0.366798 Hz
AQ 1.3631988 sec
RG 2050
DW 20.800 usec
DE 6.50 usec
TE 298.0 K
D1 2.00000000 sec
D11 0.03000000 sec
TD0 1
======== CHANNEL f1 ========
NUC1 13C
P1 8.75 usec
PL1 -2.00 dB
PL1W 58.91986084 W
SFO1 100.6001970 MHz
======== CHANNEL f2 ========
CPDPRG2 waltz16
NUC2 1H
PCPD2 80.00 usec
PL2 0.00 dB
PL12 15.78 dB
PL13 19.00 dB
PL2W 9.74611950 W
PL12W 0.25753233 W
PL13W 0.12269637 W
SFO2 400.0416002 MHz
SI 65536
SF 100.5901380 MHz
WDW EM
SSB 0
LB 1.00 Hz
GB 0
PC 1.40

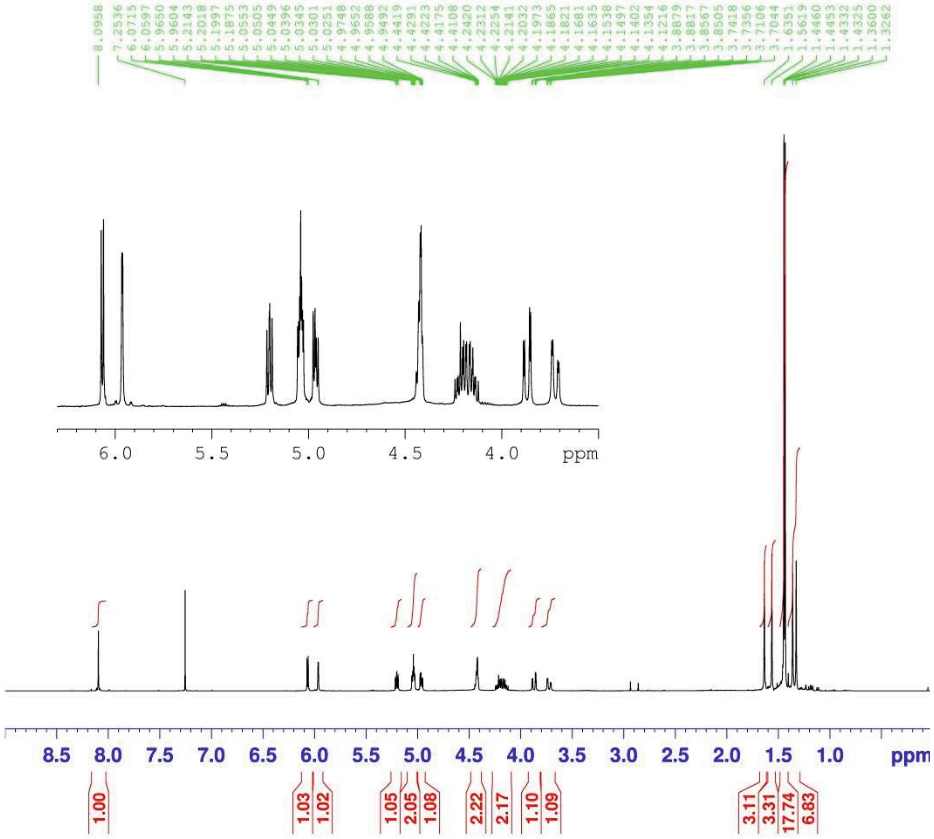

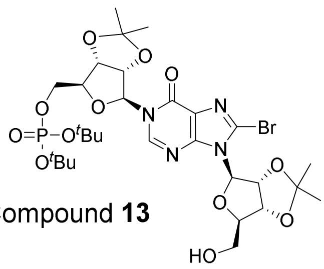

Compound **13**

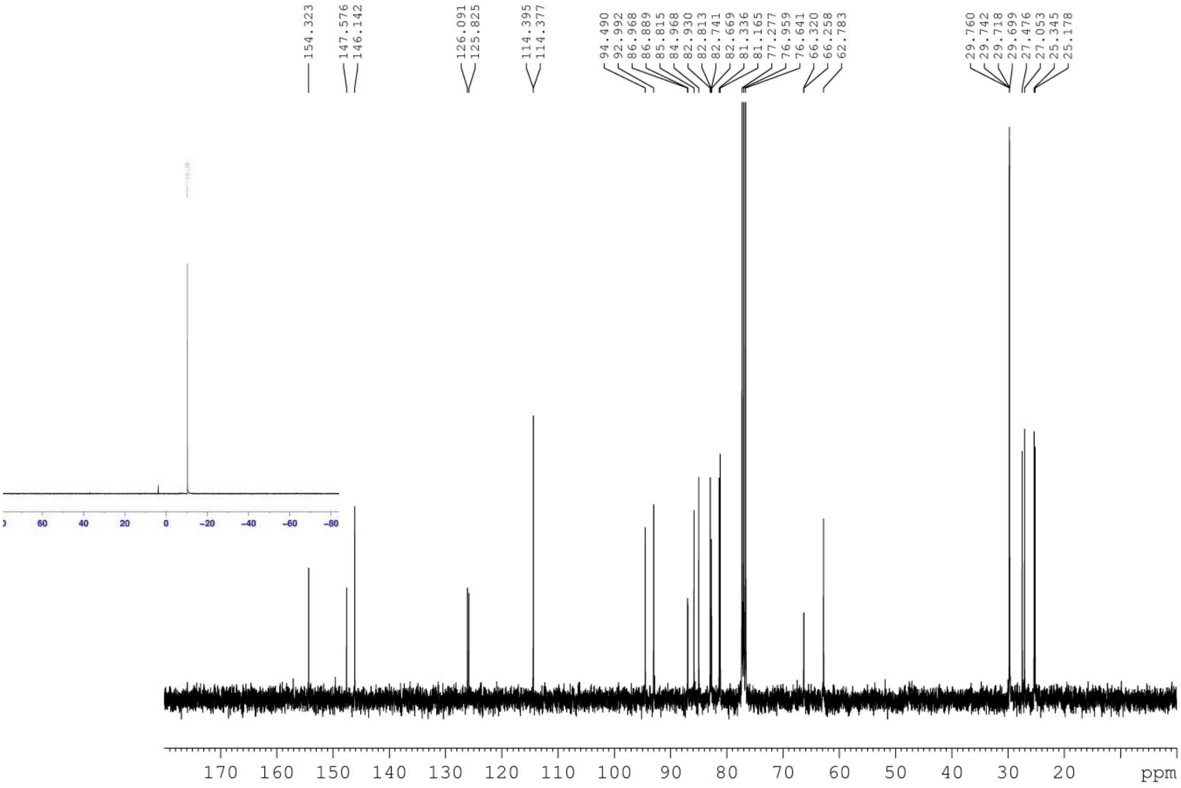


```
NAME       Nov15-2011-JMS18744
EXPNO                  12
PROCNO                  1
Date_            20111116
Time                 5.49
INSTRUM          AVIII400
PROBHD   5 mm PABBO BB-
PULPROG           zgpg30
TD                  65536
SOLVENT             CDCl3
NS                    512
DS                      4
SWH             24038.461 Hz
FIDRES           0.366798 Hz
AQ              1.3631988 sec
RG                   1820
DW                 20.800 usec
DE                   6.50 usec
TE                  298.0 K
D1             2.00000000 sec
D11            0.03000000 sec
TD0                     1

======== CHANNEL f1 ========
NUC1                  13C
P1                   8.75 usec
PL1                 -2.00 dB
PL1W          58.91986084 W
SFO1          100.6001970 MHz

======== CHANNEL f2 ========
CPDPRG2           waltz16
NUC2                   1H
PCPD2               80.00 usec
PL2                  0.00 dB
PL12                15.78 dB
PL13                19.00 dB
PL2W           9.74611950 W
PL12W          0.25753233 W
PL13W          0.12269637 W
SFO2          400.0416002 MHz
SI                  65536
SF            100.5901380 MHz
WDW                    EM
SSB                     0
LB                   1.00 Hz
GB                      0
PC                   1.40
```

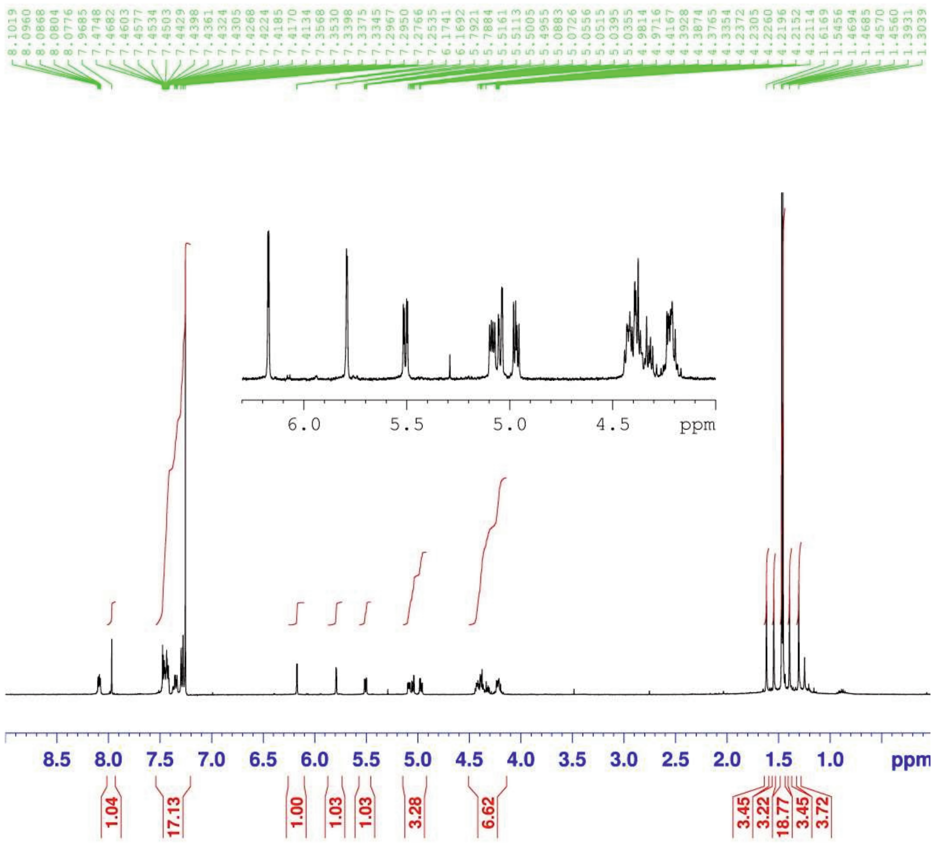

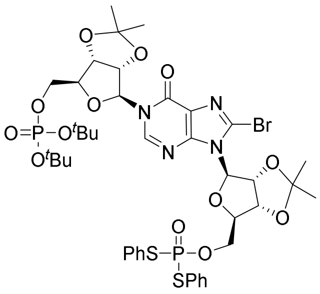

Compound **14**

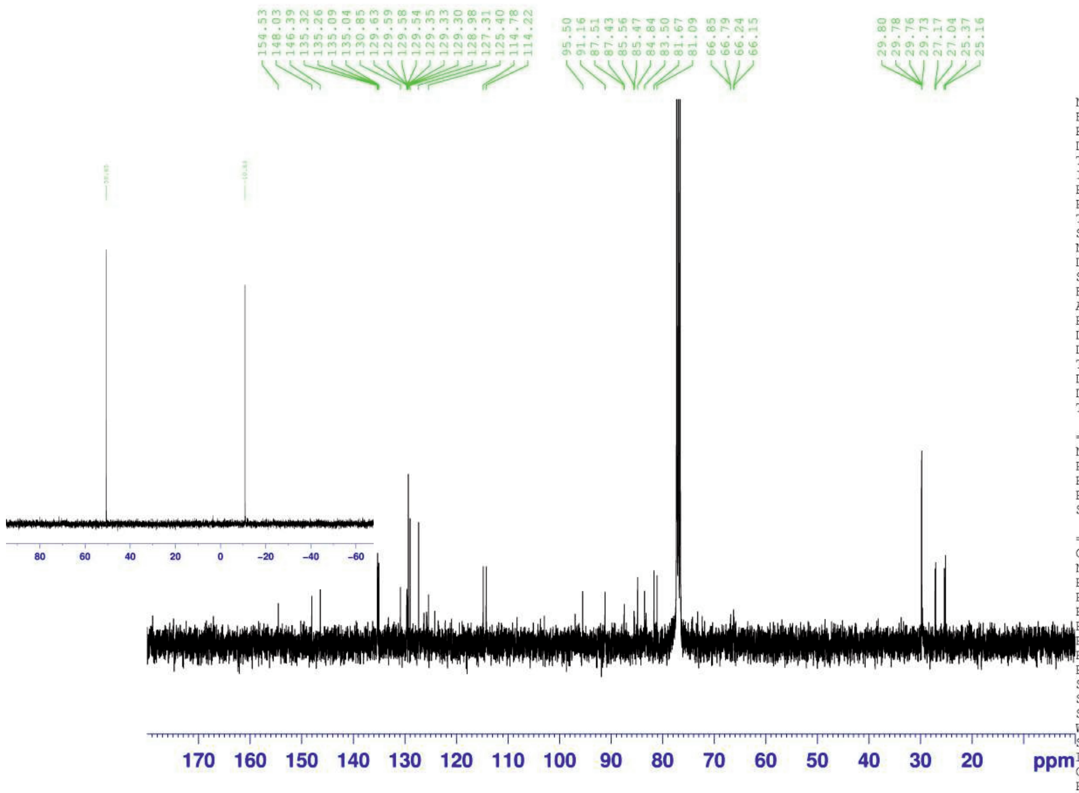

NAME Dec06-2011-JMS19267
EXPNO 12
PROCNO 1
Date_ 20111207
Time 7.00
INSTRUM AVIII400
PROBHD 5 mm PABBO BB-
PULPROG zgpg30
TD 65536
SOLVENT CDCl3
NS 2048
DS 4
SWH 24038.461 Hz
FIDRES 0.366798 Hz
AQ 1.3631988 sec
RG 1820
DW 20.800 usec
DE 6.50 usec
TE 298.0 K
D1 2.00000000 sec
D11 0.03000000 sec
TD0 1

======== CHANNEL f1 ========
NUC1 13C
P1 8.75 usec
PL1 -2.00 dB
PL1W 58.91986084 W
SFO1 100.6001970 MHz

======== CHANNEL f2 ========
CPDPRG2 waltz16
NUC2 1H
PCPD2 80.00 usec
PL2 0.00 dB
PL12 15.78 dB
PL13 19.00 dB
PL2W 9.74611950 W
PL12W 0.25753233 W
PL13W 0.12269637 W
SFO2 400.0416002 MHz
SI 65536
SF 100.5901380 MHz
WDW EM
SSB 0
LB 1.00 Hz
GB 0
PC 1.40

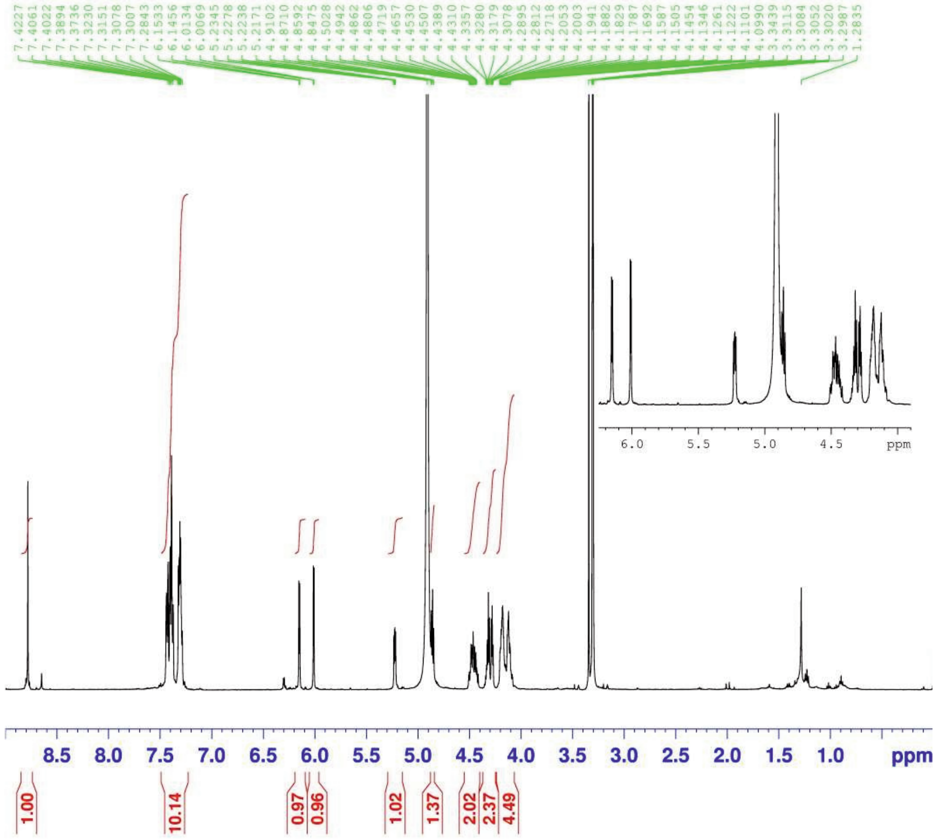

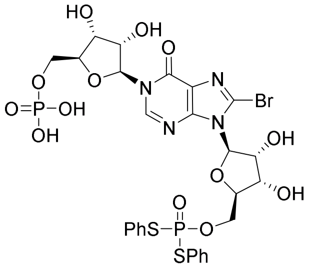

Compound **15**

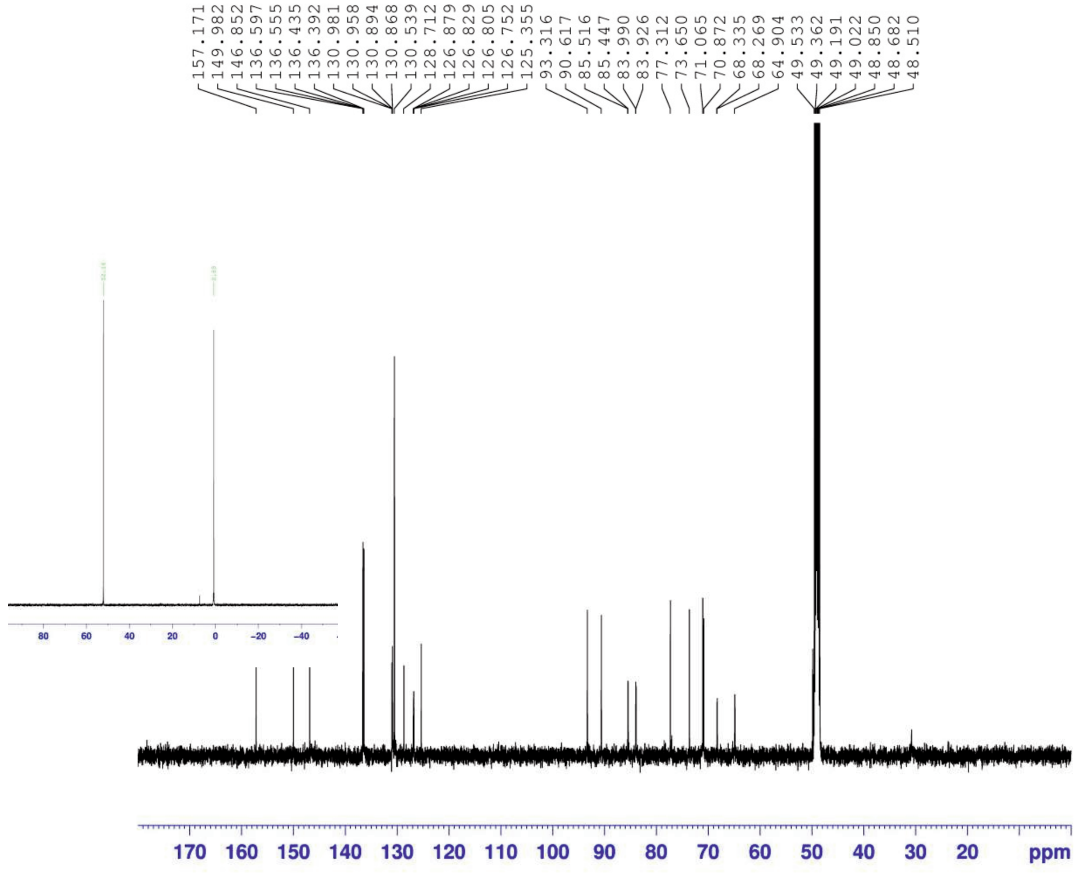

```
NAME          JMS608TT22-28
EXPNO                    11
PROCNO                    1
Date_              20111214
Time                  11.25
INSTRUM               spect
PROBHD     5 mm PABBO BB-
PULPROG             zgpg30
TD                   65536
SOLVENT               MeOD
NS                    4386
DS                       4
SWH             29761.904 H
FIDRES           0.454131 H
AQ              1.1010548 s
RG                    2050
DW                  16.800 u
DE                    8.43 u
TE                   293.1 K
D1              2.00000000 s
D11             0.03000000 s
TD0                      1

======== CHANNEL f1 =====
NUC1                   13C
P1                    9.50 u
PL1                  -0.51 d
PL1W           99.92730713 W
SFO1           125.7703643 M

======== CHANNEL f2 =====
CPDPRG2            waltz16
NUC2                    1H
PCPD2                80.00 u
PL2                  -0.12 d
PL12                 17.94 d
PL13                 21.00 d
PL2W           19.35150909 W
PL12W           0.30249262 W
PL13W           0.14952536 W
SFO2           500.1320005 M
SI                   32768
SF             125.7576104 M
WDW                     EM
SSB                      0
LB                    1.00 H
GB                       0
PC                    1.40
```

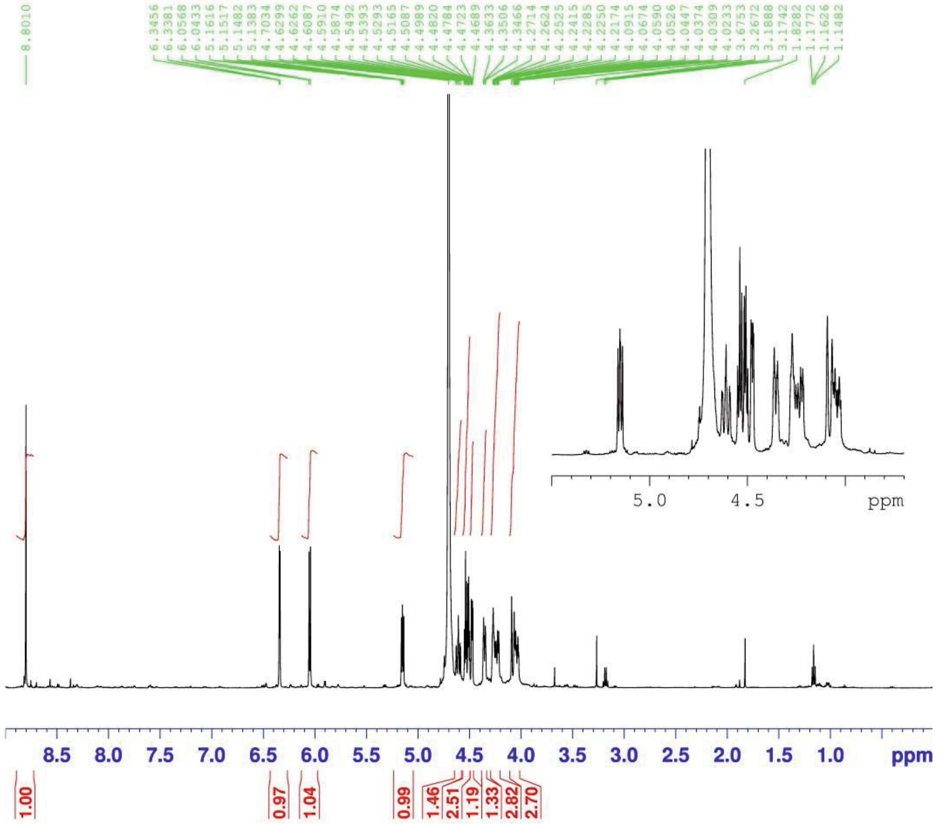

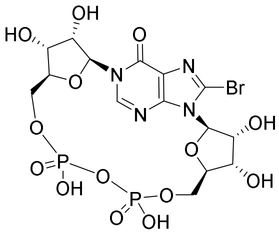

Compound **6**

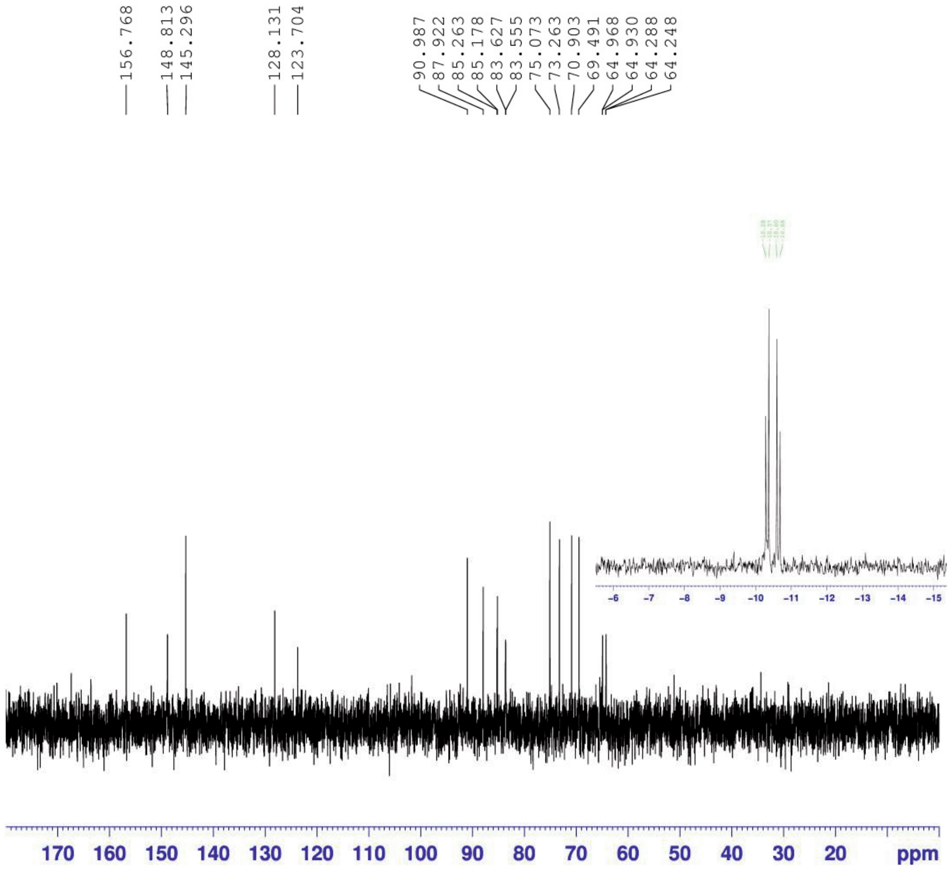

```
NAME       JMS 659 HPLC Na s
EXPNO                    19
PROCNO                    1
Date_              20120305
Time                   9.14
INSTRUM               spect
PROBHD    5 mm PABBO BB-
PULPROG              zgpg30
TD                    65536
SOLVENT                 D2O
NS                     4096
DS                        4
SWH               29761.904 Hz
FIDRES             0.454131 Hz
AQ                1.1010548 se
RG                     2050
DW                   16.800 us
DE                     8.43 us
TE                    298.1 K
D1               2.00000000 se
D11              0.03000000 se
TD0                       1

======== CHANNEL f1 =======
NUC1                    13C
P1                     9.50 us
PL1                   -0.51 dB
PL1W            99.92730713 W
SFO1            125.7703643 MH

======== CHANNEL f2 =======
CPDPRG2             waltz16
NUC2                     1H
PCPD2                 80.00 us
PL2                   -0.12 dB
PL12                  17.94 dB
PL13                  21.00 dB
PL2W            19.35150909 W
PL12W            0.30249262 W
PL13W            0.14952536 W
SFO2            500.1320005 MH
SI                    32768
SF              125.7577890 MH
WDW                      EM
SSB                       0
LB                     2.00 Hz
GB                        0
PC                     1.40
```

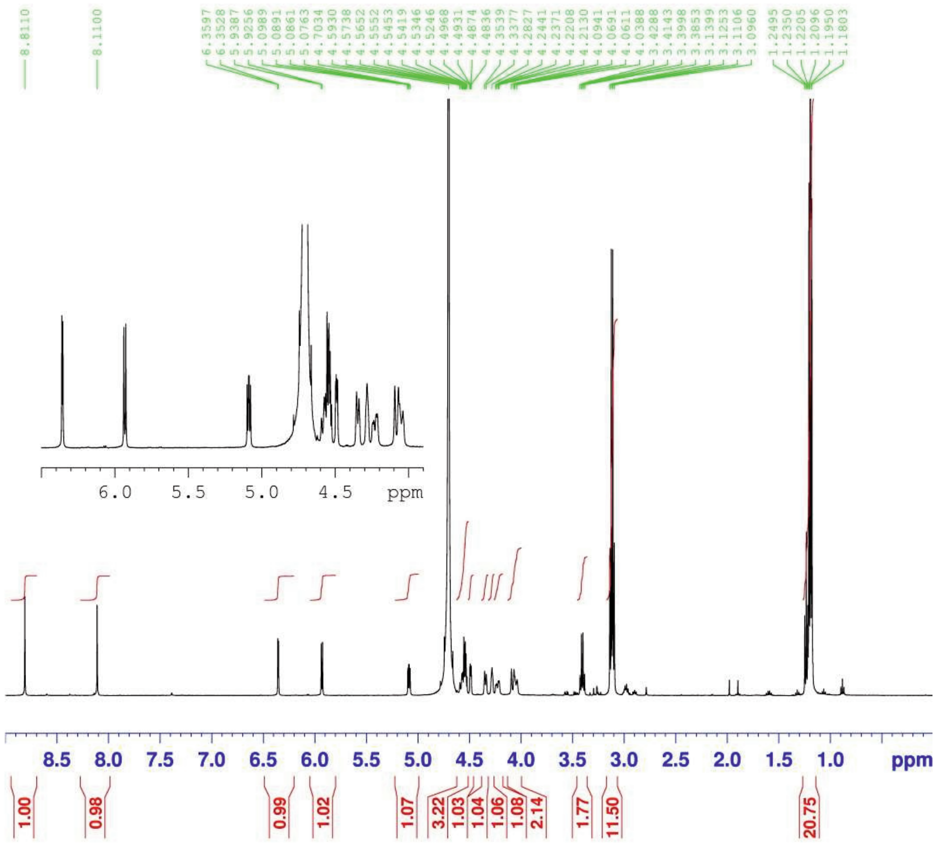

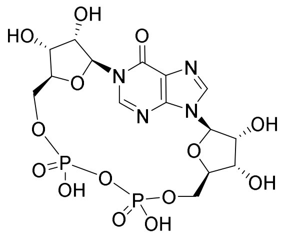

Compound **5**

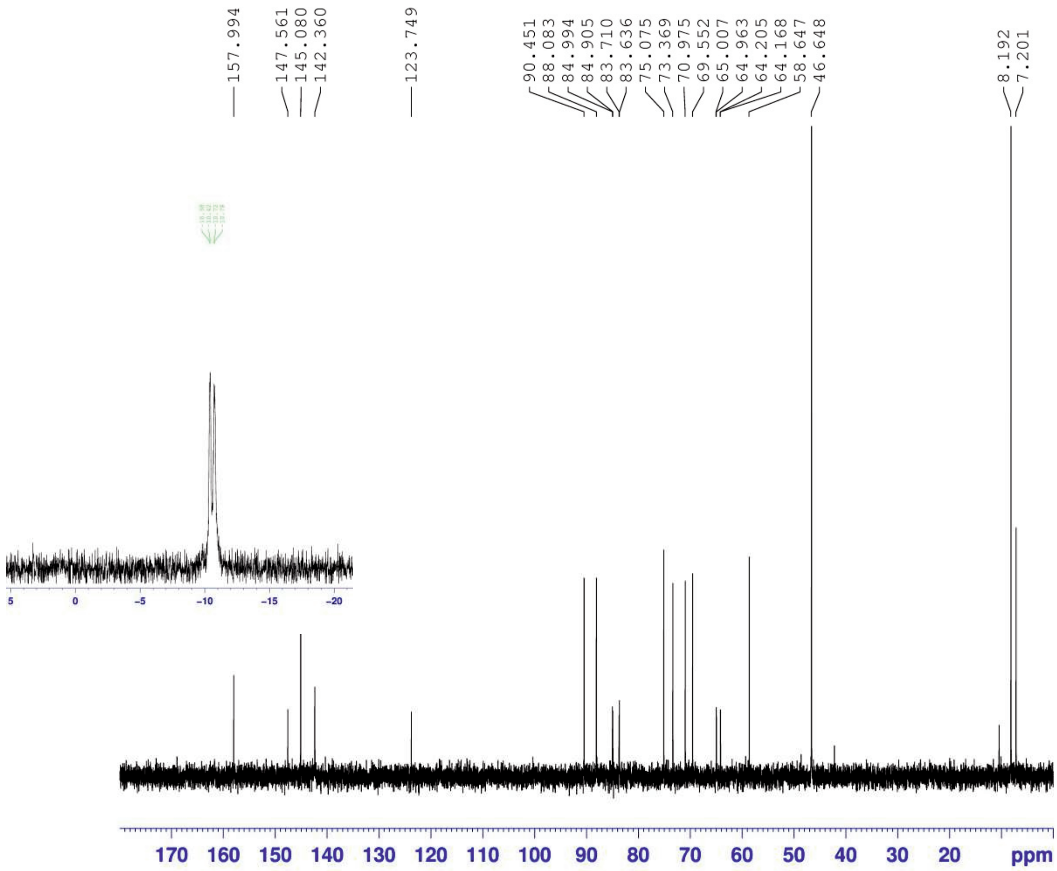


```
NAME        Apr13-2012-JMS68
EXPNO                     11
PROCNO                     1
Date_               20120415
Time                   22.56
INSTRUM                spect
PROBHD     5 mm PABBO BB-
PULPROG              zgpg30
TD                     65536
SOLVENT                  D2O
NS                     40000
DS                         4
SWH                29761.904 H
FIDRES              0.454131 H
AQ                 1.1010548 s
RG                      2050
DW                    16.800 u
DE                      8.43 u
TE                     298.0 K
D1                2.00000000 s
D11               0.03000000 s
TD0                        1

======== CHANNEL f1 =====
NUC1                     13C
P1                      9.50 u
PL1                    -0.51 d
PL1W             99.92730713 W
SFO1             125.7703643 M

======== CHANNEL f2 =====
CPDPRG2              waltz16
NUC2                      1H
PCPD2                  80.00 u
PL2                    -0.12 d
PL12                   17.94 d
PL13                   21.00 d
PL2W             19.35150909 W
PL12W             0.30249262 W
PL13W             0.14952536 W
SFO2             500.1320005 M
SI                     32768
SF               125.7577890 M
WDW                       EM
SSB                        0
LB                      1.00 H
GB                         0
PC                      1.40
```

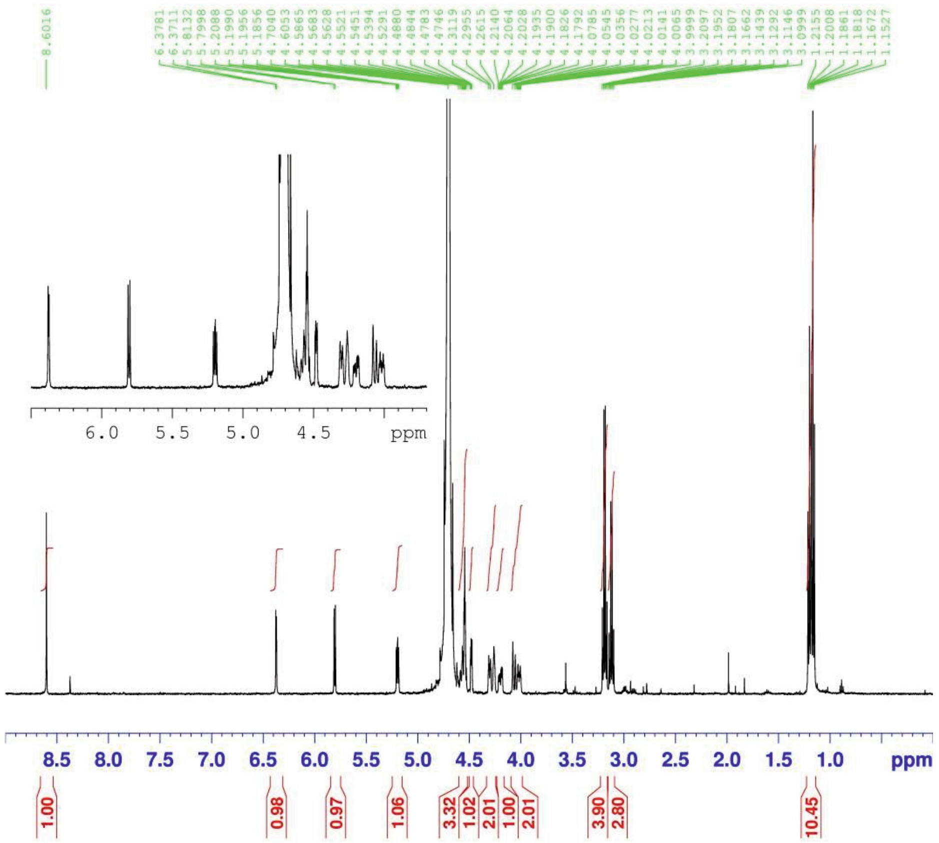

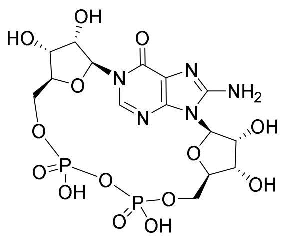

Compound **7**

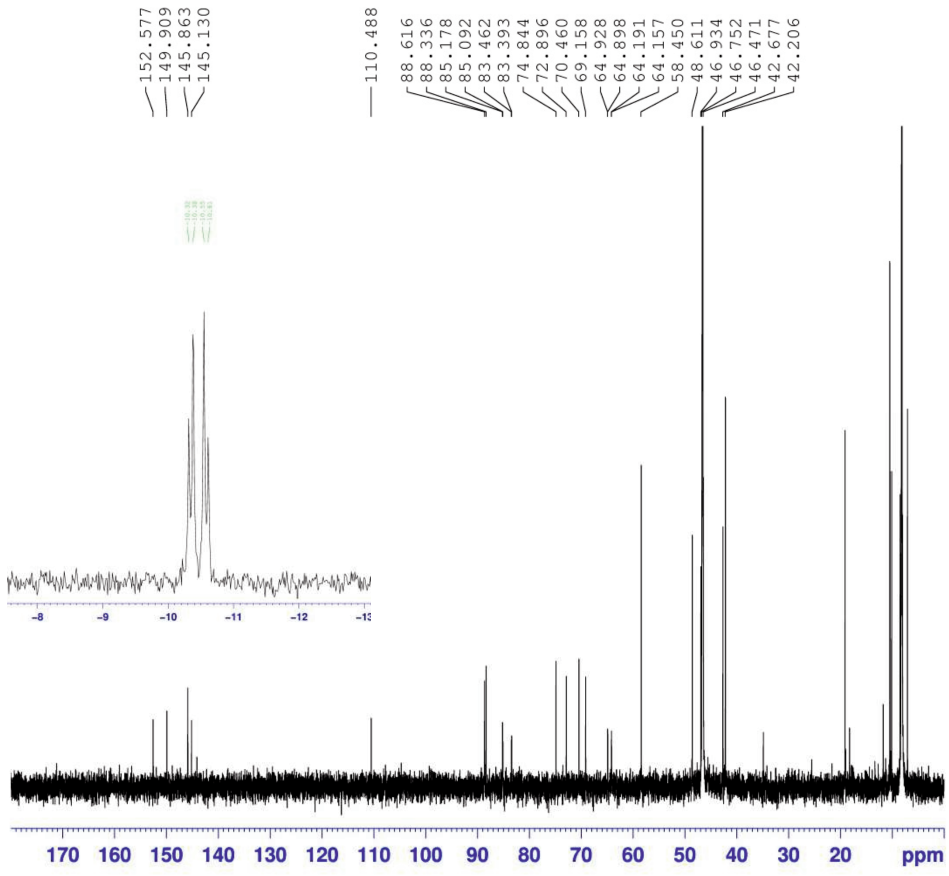

NAME JMS781ie30-36
EXPNO 22
PROCNO 1
Date_ 20130209
Time 11.36
INSTRUM spect
PROBHD 5 mm PABBO BB-
PULPROG zgpg30
TD 65536
SOLVENT D2O
NS 20480
DS 4
SWH 29761.904
FIDRES 0.454131
AQ 1.1010548
RG 2050
DW 16.800
DE 8.43
TE 298.0 K
D1 2.00000000
D11 0.03000000
TD0 1

======== CHANNEL f1 [illegible]
NUC1 13C
P1 9.50
PL1 -0.51
PL1W 99.92730713 W
SFO1 125.7703643

======== CHANNEL f2 [illegible]
CPDPRG2 waltz16
NUC2 1H
PCPD2 80.00
PL2 -0.12
PL12 17.94
PL13 21.00
PL2W 19.35150909 W
PL12W 0.30249262 W
PL13W 0.14952536 W
SFO2 500.1320005
SI 32768
SF 125.7577890
WDW EM
SSB 0
LB 1.00
GB 0
PC 1.40
